# A horizontal connectivity mode in coastal oceans: transport overrides stratification to govern microbiome network stability

**DOI:** 10.1128/aem.00448-26

**Published:** 2026-05-27

**Authors:** Yongzheng Peng, Jinling Wu, Xiaoli Zhang, Xiaoxiao Wang, Bo Wang, Chuyu Zhang, Can Wang, Honglei Zhang, Feilong Liu, Kaiyue Lian, Yi Li, Qian Liu, Hualong Wang

**Affiliations:** 1 College of Marine Life Sciences, Institute of Evolution and Marine Biodiversity, Frontiers Science Center for Deep Ocean Multispheres and Earth System, Ocean University of China 506915 , Qingdao, China; 2 UMT-OUC Joint Center for Marine Studies, Qingdao, China; 3 College of Safety and Environmental Engineering, Shandong University of Science and Technology 74789, Qingdao, China; 4 Sinopec Key Laboratory of MEOR, Petroleum Engineering Technology Research Institute, Shengli Oilfield Company, SINOPEC 12591, Dongying, China; 5 Marine Science Research Institute of Shandong Province (National Oceanographic Center, Qingdao) 639167 , Qingdao, China; University of Delaware, Lewes, Delaware, USA

**Keywords:** horizontal gradients, vertical gradients, community connectivity, network stability, coastal oceans

## Abstract

Coastal ecosystems are critical biogeochemical reactors and biodiversity reservoirs, yet the mechanisms governing the community connectivity and stability of their foundational microbiomes remain poorly understood, particularly in anthropogenically influenced coastal oceans. Here, we systematically analyzed prokaryotic and microeukaryotic communities along both vertical and horizontal gradients in the Bohai-Yellow Sea, a model anthropogenically influenced and multi-stressed coastal ocean. We found that horizontal gradients, primarily driven by distance to coastline and latitudinal transitions, surpassed vertical gradients as the principal force governing microbial community assembly, connectivity, and stability. These horizontal factors overwhelmingly shaped microbial diversity, niche breadth, and biogeographic patterns, with prokaryotes displaying broader environmental adaptability and stronger cross-regional dispersal potential than microeukaryotes. Consequently, prokaryotes maintained higher spatial connectivity than microeukaryotes across both dimensions. This prokaryotic connectivity advantage was most pronounced within intermediate water masses. In contrast, vertical connectivity weakened with intensified water column stratification from the well-mixed Bohai Sea to the hydrographically complex South Yellow Sea. Horizontal gradients directly steered microbial co-occurrence network properties, driving a depth-dependent decline in network complexity and stability. This regional disparity was further underscored by distinct stability trade-offs: the semi-enclosed Bohai Sea fostered robust, modular networks, whereas the Yellow Sea systems formed highly connected but less modular, and thus more vulnerable, network architectures. Our findings reveal a dominant horizontal connectivity mode in coastal microbiomes, establishing a mechanistic link between large-scale environmental gradients and microbial network stability. This is a crucial advance for predicting ecological responses to anthropogenic and climate perturbations in vulnerable coastal zones.

**IMPORTANCE:**

Coastal microbial communities drive global biogeochemical cycles, yet the principles governing their large-scale connectivity and microbial network stability remain elusive, particularly in anthropogenic disturbances regions. Focusing on the Bohai-Yellow Sea system, we establish that horizontal transport processes, modulated by land-sea exchange and latitudinal gradients, override vertical stratification as the dominant force structuring microbial assembly and interaction networks. We demonstrate that prokaryotes possess a stronger horizontal dispersal advantage than microeukaryotes, sustaining higher connectivity through intermediate water layers. This horizontal connectivity governs microbial network stability. Networks shift from robust, prokaryote-driven modular architectures in shallow coastal waters to fragile, microeukaryote-dominated patterns in deeper, stratified regions. These findings define a “horizontal connectivity mode” as a central organizing principle for coastal microbiomes, moving beyond descriptive biogeography to provide a mechanistic framework for predicting community resilience to anthropogenic and climate forcing.

## INTRODUCTION

The marine ecosystem, as the largest on Earth, regulates global energy flow and biogeochemical cycling through intricate biological networks (1). Microbial communities are fundamental to these processes, contributing 52%–67% of total marine primary production (2–4). Environmental heterogeneity across horizontal and vertical dimensions profoundly impacts microbial community dynamics and ecological functions (5, 6), particularly in coastal oceans with pronounced terrestrial and anthropogenic influences (7, 8).

Environmental drivers for shaping microbiome dynamics and functions are often highly complex and spatially heterogeneous across coastal oceans along horizontal and vertical dimensions (9–16). In transitional systems like estuaries, horizontal gradients in salinity and temperature often emerge as primary drivers for microbiome distributions (17–19). Yet studies in the Rongjiang River estuary reveal that vertical variations in water depth could shape community stability under specific conditions (20, 21). In the coastal East China Sea, strong terrestrial inputs create pronounced horizontal gradients in shaping bacterioplankton communities (22), while fishing activities in the northern South China Sea amplify the role of water depth in structuring diatom communities (23). Critically, the intensity of anthropogenic pressures typically diminishes along both vertical depth and horizontal offshore gradients (24, 25). These results suggest that the relative importance of horizontal vs vertical gradients to shape microbiomes appears to be highly variable across different coastal ecosystems.

As a semienclosed body of water serving as the estuary for multiple rivers, including the Yellow River, the biogeochemical cycles of the Bohai Sea are significantly influenced by terrestrial inputs and anthropogenic activities (26–29). However, how microbial community connectivity and network stability change along horizontal vs vertical gradients in the Bohai Sea and Yellow Sea remains unclear. Here, we define connectivity as the proportion of ASVs shared with source habitats (30) and stability as the structural integrity of microbial co-occurrence networks (31–33). One hundred thirty-eight samples were collected in the Bohai-Yellow Sea to test the hypotheses that (i) horizontal gradients exert a stronger influence on microbiome assembly and network organization than vertical gradients and (ii) microeukaryotes exhibit greater sensitivity to these spatial variations than prokaryotes. By testing these hypotheses, this work sheds light on the mechanisms driving microbial dynamics and associations in coastal oceans subject to intense terrestrial and anthropogenic pressures.

## MATERIALS AND METHODS

### Sample collection

To compare the spatial connectivity of prokaryotic and microeukaryotic communities across the Bohai Sea (BH), North Yellow Sea (NY), and South Yellow Sea (SY) (32°–39.5°N, 119.9°–124.5687°E) (Fig. S2), a total of 138 water samples (69 for prokaryotes and 69 for microeukaryotes) were collected from 12–28 April 2023. Seawater samples were collected from surface, middle, and bottom layers (classified as BHS/BHM/BHB, NYS/NYM/NYB, SYS/SYM/SYB) using a SeaBird CTD. We stratified the water column using CTD-derived density profiles. Potential density was calculated from temperature and salinity using the Gibbs SeaWater equation, and vertical density gradients were assessed between consecutive depths (Fig. S3). Stations with a pycnocline (density gradient >0.01 kg m^−3^ m^−1^) had layers defined by its boundaries. For stations lacking a pycnocline, layers were assigned by relative depth: surface (0%–30%), middle (30%–70%), and bottom (70%–100%) (34) (Table S1). For dissolved inorganic nutrients (NO_3_^−^-N, NO_2_^−^-N, PO_4_^3−^-P, NH_4_^+^, SiO_3_^2−^-Si), 50-mL seawater was collected and stored at −20°C prior to quantification via continuous-flow colorimetry (Bran + Luebbe AutoAnalyzer 3) under JGOFS protocols. Seawater was sequentially filtered through 3-μm and 0.22-μm polycarbonate membranes. Both filters were combined and stored at −80°C to represent the total microbial communities in each sample. Total DNA was subsequently extracted from the combined filters.

### DNA extraction and sequencing

Genomic DNA was isolated through lysozyme-SDS-phenol/chloroform protocols and amplified with uniquely indexed primers targeting the 16S and 18S ribosomal RNA (rRNA) gene regions. The V3-V4 hypervariable region of the 18S rRNA gene was amplified with primer pair 528F/706R (35), while the V4 domain of prokaryotic 16S rRNA was targeted using universal primers 515F/806R (36, 37). High-resolution sequencing was performed on an Illumina Nova6000 platform (250-bp paired-end) at Magigene Biotechnology (Guangzhou, China).

Processing of the raw sequences was conducted with the QIIME 2 (v2021.4) platform, including (i) quality filtering and clustering into amplicon sequence variants (ASVs), (ii) taxonomic classification against SILVA 138-99 using the q2-feature-classifier Naïve Bayes plugin (99% confidence), and (iii) removal of low-abundance ASVs (<5 total observations or <2 samples). Domain-specific filtering excluded unclassified sequences for microeukaryotes and chloroplast-associated reads and unassigned reads for prokaryotes. Phylogenetic reconstruction utilized maximum-likelihood evolutionary placement algorithms.

To standardize sequencing effort, rarefaction analysis was conducted by subsampling 67,000 sequences per sample for both domains. The final curated data set comprised 2,119 microeukaryotic and 2,438 prokaryotic ASVs, with rarefaction curves confirming adequate sequencing coverage (Fig. S1).

### Diversity and dynamic analysis of microbial communities

Alpha- and beta-diversity metrics were calculated in QIIME 2 (v2021.4) using rarefied ASV tables. Alpha-diversity included the observed ASVs, Faith’s PD, Pielou’s J, Simpson (1-λ), and Shannon (H'). The spatial distribution heatmap of species richness was drawn using Ocean Data View (v5.6.2). Beta-diversity incorporated Bray-Curtis, Jaccard, and UniFrac (weighted/unweighted) metrics based on a FastTree2-derived 16S/18S rRNA phylogeny.

Microbial communities were analyzed using NMDS ordination (Bray-Curtis dissimilarity; Hellinger-transformed ASVs; stress <0.2) in R 4.1.1 (vegan v2.6-4). Basin differentiation (BH, NY, SY) was tested via ANOSIM (global R-statistic; 9,999 permutations). For paired environmental variables, the coefficient of variation (CV, %) was calculated to quantify horizontal and vertical gradients, with the dominance of horizontal gradients verified via 9,999 permutation tests. Associations between microbial communities and environmental variables were assessed by Mantel tests (10,000 permutations, FDR < 0.05) for community dissimilarity against spatial and normalized environmental gradients. Intercorrelations among environmental variables were assessed using Spearman’s (Holm-adjusted *P* < 0.05). Distance-based redundancy analysis (dbRDA) was used to evaluate environmental effects on microbial communities. After standardization and collinearity filtering (VIF < 10), variable significance was assessed via marginal permutation tests, with independent effects (adjusted *R*^2^) quantified through hierarchical partitioning.

### Spatial connectivity and assembly processes of microbial communities

We assessed spatial gradients in the Bohai-Yellow Sea by calculating distances to coastline using the GSHHG data set and classifying stations as nearshore (<20 nautical miles), mid-distance (20–60 nautical miles), or offshore (>60 nautical miles) based on anthropogenic influence (38). To evaluate connectivity along gradients of decreasing disturbance, we performed three directional source-tracking analyses. These included vertical (across water layers within each region), horizontal (from BH to NY to SY for each layer), and nearshore-offshore. Connectivity from a source to a target was defined as the percentage of ASVs in the target that were also detected in the source along each of these spatial gradients, providing a directional, presence-based measure of microbial dispersal (30). Statistical significance was assessed using two-proportion z-tests with Yates’ correction and BH adjustment. A sensitivity analysis excluding rare ASVs (relative abundance < 0.01% and occurrence < 10%) was performed to confirm robustness.

The niche breadth of microbiomes was quantified in R (v4.1.1) using the “spaa” package’s. The assembly processes of prokaryotic and microeukaryotic communities were calculated by neutral community models (39) (“minpack.lm” package in R 4.1.1). The abundance-weighted β mean nearest taxon distance (β-MNTD) between ASVs was computed using the comdistnt function in R package “picante” (v1.8.2) (40). The β nearest taxon index (β-NTI) was then calculated by standardizing the observed β-MNTD values against the null distribution, identifying dominant assembly processes (41–43). For comparisons with |β-NTI| ≤ 2, we further employed the Bray-Curtis-based Raup-Crick index (RCbray) to distinguish among stochastic processes. All analyses were performed with parallel computing and fixed random seeds (set.seed = 123) to ensure reproducibility

### Co-occurrence network properties and stability analysis

We constructed cross-domain co-occurrence networks by merging prokaryotic and microeukaryotic ASV tables for each region using Random Matrix Theory (RMT)-based Molecular Ecological Network Analysis (MENA) (33, 44–46). After filtering ASVs detected in <50% of samples, we calculated Spearman correlations for all remaining pairs. Region-specific RMT thresholds (~0.85) were applied to preserve non-random signals, and the iDIRECT approach was used to minimize indirect and environmentally driven associations (33). Only pairs meeting both |*r*| ≥ threshold and *P* < 0.001 were retained as edges. Network topological properties were assessed as complexity indicators (47), with statistical significance evaluated against 1,000 degree-preserving random networks. Node roles (Network hubs, module hubs, connectors) were assigned based on within-module connectivity and participation coefficients.

Network stability was assessed through fragmentation, robustness, and vulnerability analyses (44). Fragmentation was evaluated by quantifying the reduction in network connectivity following the targeted removal of the top 5%, 10%, and 20% of nodes ranked by betweenness centrality. Robustness was determined as the proportion of nodes remaining after 50% random taxon removal, averaged across 1,000 simulation iterations. Vulnerability was characterized by the maximum node vulnerability value within the network. Together, these metrics provided a complementary evaluation of microbial network stability across the Bohai-Yellow Sea.

## RESULTS

### Community diversity and distribution of coastal microbiomes

**Fig 1:**
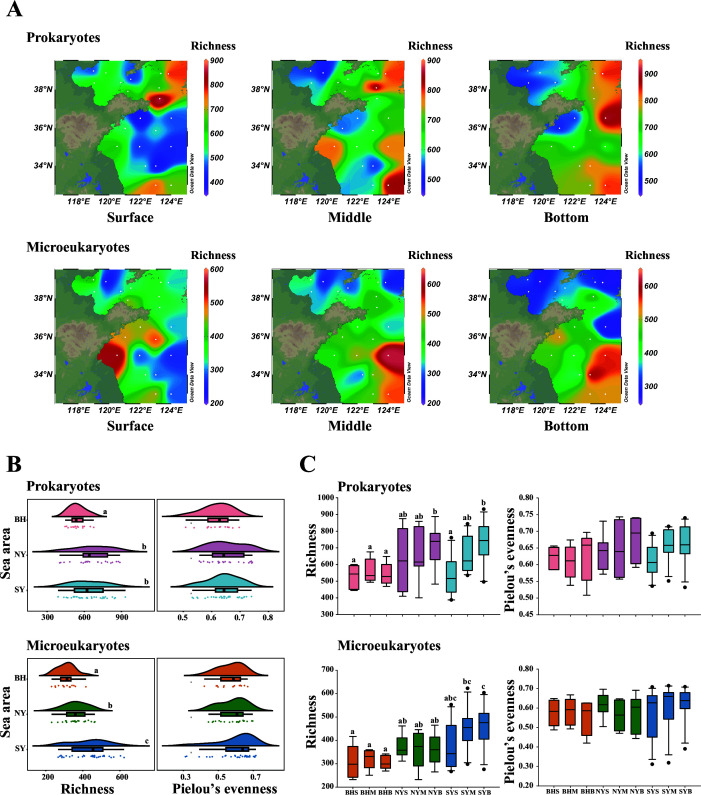
Microbial community diversity in the Bohai-Yellow Sea. (**A**) The spatial distribution heatmap of species richness in the seawater of the Bohai-Yellow Sea. (**B and C**) Richness and evenness of microbiomes along horizontal and vertical gradients in the Bohai-Yellow Sea.

Significant variations in microeukaryotic community richness were observed across the BH (*P* < 0.05), NY, and SY, whereas prokaryotic richness was only significantly different between the Bohai Sea and Yellow Sea (*P* < 0.05) (Fig. 1B). Vertical stratification patterns diverged regionally. Bohai Sea prokaryotic and eukaryotic microbial communities showed no significant depth-related differences (*P* > 0.05) (Fig. 1C). NY prokaryotes exhibited significant surface-middle-bottom stratification but absent in microeukaryotes. The richness of SY communities demonstrated significant depth-dependent increases, especially for those of prokaryotes. However, both prokaryotic and microeukaryotic communities showed no significant differences in evenness (*P* > 0.05) across spatial gradients (Fig. 1B and C). These results suggested that spatial gradients significantly shaped microbial community diversity, especially for the horizontal gradients.

**Fig 2:**
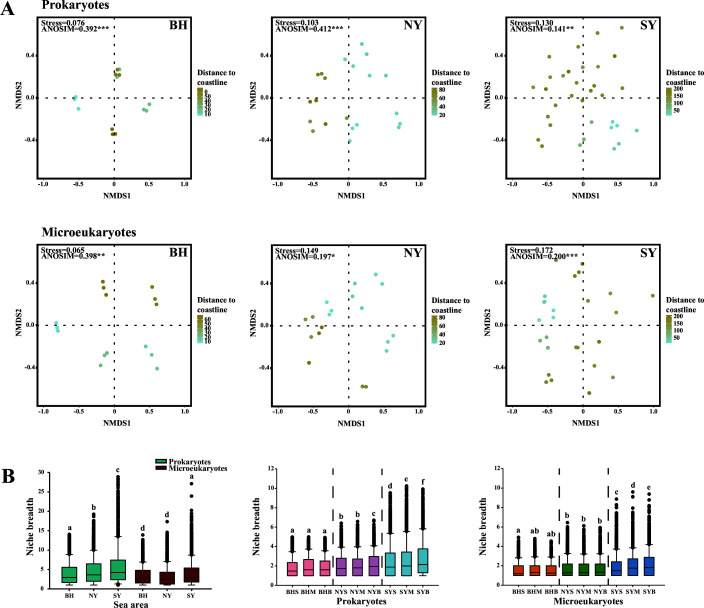
Microbial community distribution in the Bohai-Yellow Sea. (**A**) Non-metric multidimensional scaling (NMDS) ordination of seawater microbial communities based on Bray-Curtis distance along distance to coastline. (**B**) Environmental niche breadth of microbiomes in the Bohai-Yellow Sea across spatial gradients. Different letters above the bars indicate significant differences among microbial communities (*P* < 0.05), while shared letters indicate no significant difference.

Horizontal gradients significantly altered microbial communities composition. Mantel tests identified that distance to coastline is the primary spatial factor for driving microbial communities distribution (Fig. 2A and Table 1). Additionally, BH-SY showed stronger microbial communities distribution differences than BH-NY at the same layer. The vertical differentiation of prokaryotic communities showed significant variation only in the SY (between surface and bottom layers, *P* = 0.03; Fig. S5 and Table S2). Such differentiation was not observed in the BH or NY (*P* > 0.05) (Fig. S5 and Table S2). These results indicated that horizontal gradients outweigh vertical gradients in shaping microbial community distribution, with the effect of vertical gradients only observed in deeper region, such as the SY.

**TABLE 1:** Mantel test assessing the correlation between microbial community composition and environmental factors in the Bohai-Yellow Sea^*a*^

Environmental variable	Bohai Sea				North Yellow Sea				South Yellow Sea			
	Prokaryotes		Microeukaryotes		Prokaryotes		Microeukaryotes		Prokaryotes		Microeukaryotes	
	*r*	*P*	*r*	*P*	*r*	*P*	*r*	*P*	*r*	*P*	*r*	*P*
Longitude	0.233	0.014*	0.541	0.001***	0.028	0.327	−0.002	0.498	0.060	0.186	0.037	0.265
Latitude	−0.059	0.589	0.222	0.017*	0.024	0.337	0.084	0.156	0.124	0.085	0.106	0.053
Distance to coastline	0.238	0.035*	0.474	0.001***	0.501	0.001***	0.422	0.001***	0.181	0.031*	0.085	0.104
Depth	−0.143	0.934	−0.114	0.898	−0.015	0.537	−0.102	0.930	0.046	0.191	−0.033	0.694
Chl a	−0.018	0.483	0.133	0.086	−0.021	0.582	0.194	0.019*	−0.042	0.591	0.119	0.093
Temperature	0.271	0.01**	0.521	0.001***	0.268	0.007**	0.158	0.026*	0.178	0.009**	0.216	0.002**
Dissolved oxygen	0.255	0.053	0.323	0.007**	0.201	0.012*	0.025	0.333	0.197	0.037*	0.146	0.022*
Salinity	0.735	0.001***	0.575	0.001***	0.510	0.001***	0.324	0.001***	0.338	0.001***	0.240	0.001***
Turbidity	0.490	0.002**	0.075	0.182	0.027	0.343	0.197	0.018*	0.055	0.172	−0.080	0.788
pH	0.609	0.001***	0.521	0.001***	0.499	0.001***	0.481	0.001***	0.052	0.197	0.119	0.031*
Nitrate	0.571	0.001***	0.324	0.005**	0.160	0.041*	0.278	0.003**	0.189	0.066	−0.015	0.553
Nitrite	0.334	0.016*	0.224	0.021*	0.038	0.291	0.228	0.005**	0.180	0.08	−0.020	0.537
NH_4_^+^	0.114	0.264	0.012	0.418	0.027	0.397	0.156	0.081	0.030	0.306	−0.070	0.818
PO_4_^3−^	−0.215	0.92	0.174	0.031*	0.110	0.092	0.143	0.048*	0.166	0.092	−0.028	0.611
SiO_3_^2−^	0.623	0.001***	0.346	0.004**	−0.073	0.775	0.018	0.327	0.179	0.069	0.126	0.052

^*a*^ Asterisks indicate a significant correlation between microbial diversity and environmental factors (*, *P* < 0.05; **, *P* < 0.01; ***, *P* < 0.001).

### Community assembly of coastal microbiomes

The niche breadth of coastal microbiomes exhibited a significant clinal increase along the horizontal gradients from the BH to the NY and SY, a pattern that was particularly pronounced in prokaryotic communities (Fig. 2B). This trend was consistently observed across different depth layers (Fig. 2B and Fig. S8). Vertically, niche breadth remained largely stable in the well-mixed BH and NY but showed significant variation in the stratified water column of the SY. Collectively, these results demonstrate that the environmental resource utilization of coastal microbiomes is predominantly structured by horizontal gradients, with prokaryotes exhibiting a broader and more responsive niche width than microeukaryotes (Fig. S8).

**Fig 3:**
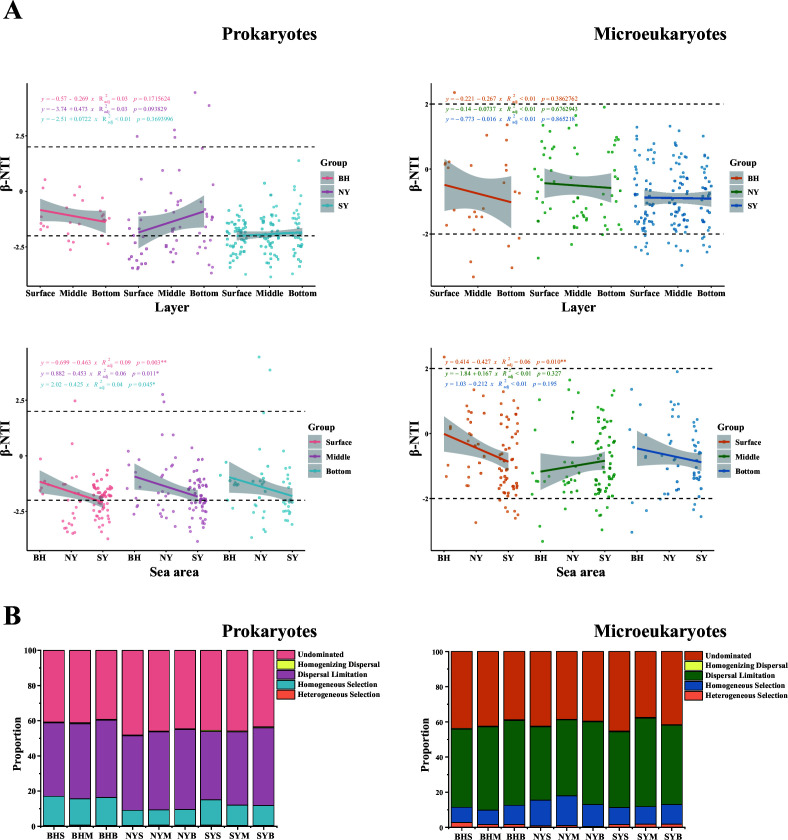
Assembly of microbial communities in the Bohai-Yellow Sea. (**A**) Beta nearest taxon index (βNTI) along vertical and horizontal gradients. (**B**) Relative contributions of ecological processes governing microbial community assembly.

Stochastic processes dominated the assembly of both prokaryotic and microeukaryotic communities across the Bohai-Yellow Seas (|β-NTI| < 2.0) (Fig. 3A), primarily driven by the combined effects of dispersal limitation and undominated (Fig. 3B). While the relative importance of deterministic processes varied significantly across geographic regions, it exhibited no significant variation with depth within any sampled region. A notable regional contrast was observed: homogeneous selection made the lowest contribution to prokaryotic assembly in the NY, whereas it exerted the strongest influence on microeukaryotic communities in the same region (Fig. 3).

Vertically, the balance of stochastic and deterministic assembly processes for both prokaryotes and microeukaryotes showed no significant differences within individual water columns (Fig. 3). Horizontally, however, the overall role of stochasticity decreased significantly from the semi-enclosed BH to the more open NY and SY, a pattern that was evident at all depth layers and more pronounced in prokaryotes (Fig. 3A; Fig. S6).

This shift in assembly mechanisms was further reflected in neutral model patterns (Fig. S6). Microbial communities in the SY exhibited the highest fit to the neutral model (*R*^2^ = 0.729 for prokaryotes; *R*^2^ = 0.631 for microeukaryotes) yet paradoxically experienced the lowest estimated dispersal rates (Nm = 1,886 for prokaryotes; Nm = 563 for microeukaryotes). In contrast, communities in the BH, characterized by the highest dispersal rates (Nm = 3,916 for prokaryotes; Nm = 961 for microeukaryotes), showed a lower model fit (*R*^2^ = 0.667 for prokaryotes; *R*^2^ = 0.560 for microeukaryotes) (Fig. S6). Notably, prokaryotic communities consistently displayed a better fit to the neutral model than microeukaryotes across all regions.

Overall, our results demonstrate that horizontal environmental gradients exert a stronger influence on microbial community assembly than vertical stratification. The overarching stochasticity, which diminishes from the BH to the SY, is primarily governed by dispersal limitation, highlighting its critical role in shaping the microbial biogeography of this coastal system.

### Co-occurrence network topology and stability

**Fig 4:**
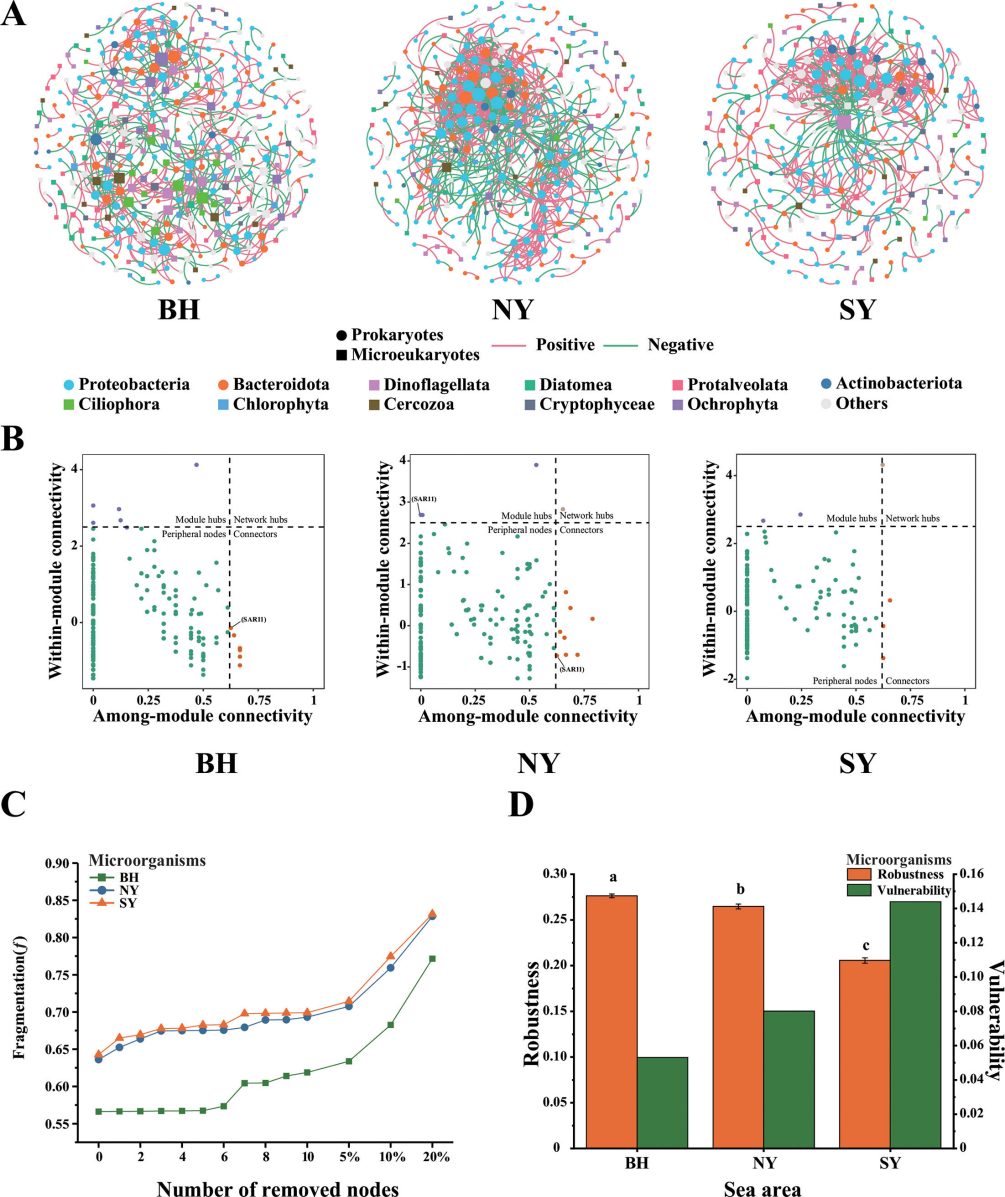
Co-occurrence network characteristics and stability of microbial communities in the Bohai–Yellow Sea. Co-occurrence network pattern (**A**), key nodes (**B**), network fragmentation (**C**), and network robustness and vulnerability (**D**) of microbial networks.

Microbial co-occurrence networks exhibited distinct topological architectures across the Bohai-Yellow Seas (Fig. 4A and Table S4), reflecting clear differences in community complexity and stability. All metrics differed significantly from null expectations, confirming non-random network structures (Table S5). The BH community networks were characterized by the highest modularity (0.783) and the lowest edge density (1.80), featuring no network hubs while 6 module hubs and 29 connected components (Fig. 4B and Table S4), and suggesting a compartmentalized and stable architecture. In contrast, the NY displayed the highest edge density (2.58) and largest number of connected components (41), yet lowest modularity (0.581), regulated by a network hub and module hubs (Fig. 4B), indicating a more complex but less organized topology. The SY featured the highest clustering coefficient (0.362) and moderate modularity (0.648), structured hierarchically around a network hub and two module hubs (Fig. 4B). Notably, the number of connected components varied regionally (BH:29, NY:41, SY:38), further underscoring systematic shifts in network integration along the horizontal gradients.

Taxonomic analysis revealed that keystone species shifted both taxonomically and functionally across regions. While prokaryotes numerically dominated network nodes, microeukaryotes, specifically Sarcinochrysidales in the NY and Dinophyceae in the SY, served as network hubs, suggesting their heightened ecological influence in the Yellow Sea systems. The SAR11 clade exhibited clear functional transitions across basins: acting as a module hub and a connector in the NY, a connector in the BH, and playing a minimal role in the SY (Fig. 4B and Table S3).

Stability assessments further highlighted these regional trade-offs. The BH network demonstrated the lowest fragmentation, vulnerability, and highest robustness, whereas the SY was the most vulnerable (Fig. 4C and D). Targeted removal of top nodes with high betweenness centrality caused the most severe fragmentation in the SY, followed by the NY, with the BH remaining largely unaffected (Fig. 4C). These results suggested microbial network architecture transitioned from a stable, modular, and prokaryote-dominated system in the semi-enclosed BH to increasingly complex yet vulnerable microeukaryote-regulated networks along the horizontal gradient toward the open Yellow Sea. This architectural shift shows how coastal microbiomes reorganize their interaction patterns in response to changing environmental forcing across regional seascapes.

### Microbial spatial connectivity across the Bohai-Yellow Sea

**Fig 5:**
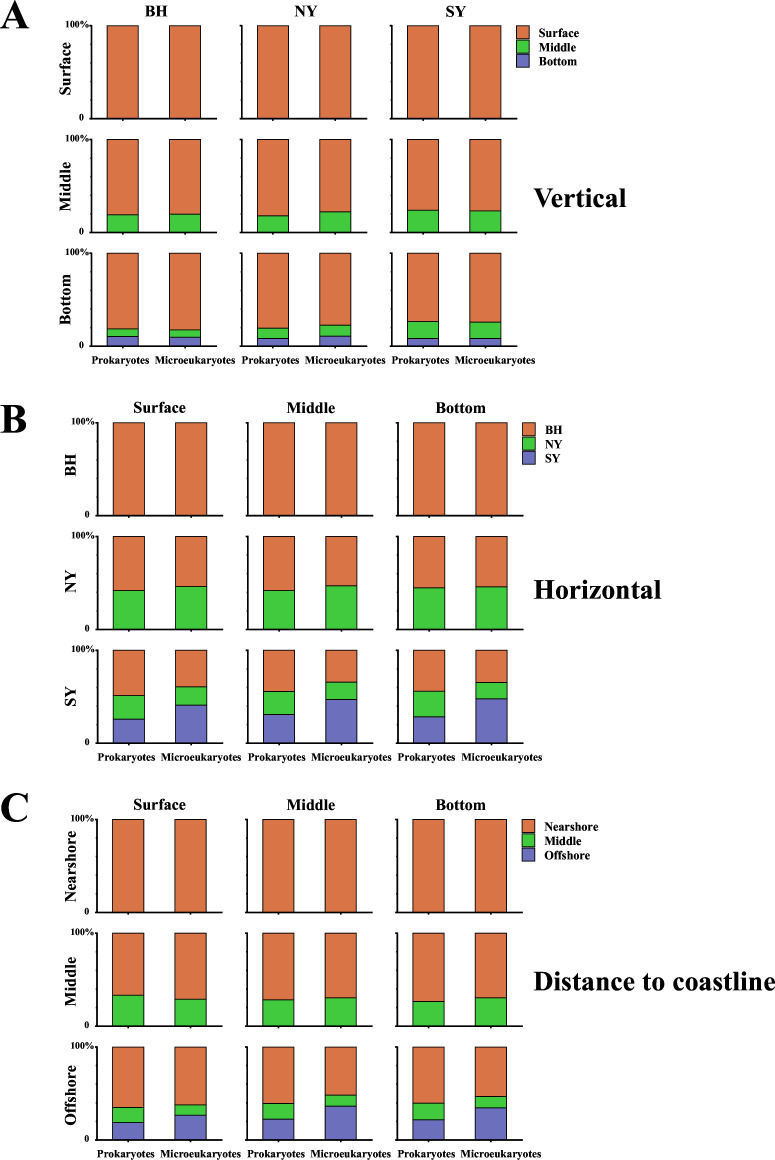
Spatial connectivity of microbial ASVs. Connectivity (%) is the percentage of ASVs in the target community that were also detected in the source community along the specified gradients. (**A**) Vertical connectivity across layers within each region. (**B**) Horizontal connectivity along the BH to NY to SY gradient for each layer. (**C**) Nearshore-offshore connectivity along the distance to coastline for each layer.

Vertical connectivity of both prokaryotic and microeukaryotic communities decreased progressively from the shallow BH (average 18 m) to the deeper SY (average 45.3 m). Connectivity values ranged from 73.45% to 81.34% for prokaryotes and 73.90% to 82.46% for microeukaryotes across regions, with both groups maintaining comparable connectivity strengths throughout the water column (Fig. 5A and Table S9).

Horizontal connectivity patterns differed markedly between prokaryotes and microeukaryotes. Prokaryotes consistently demonstrated stronger transfer capacities than microeukaryotes, both in cross-regional and coastal-offshore contexts. Their cross-regional connectivity ranged from 44.09% to 48.76%, compared to 34.24%–39.28% for microeukaryotes (Fig. 5B). Similarly, prokaryotic coastal-offshore transfer values were 60.40%–64.90%, exceeding the microeukaryotic range of 51.57%–62.23%. This prokaryotic advantage was most pronounced in intermediate water layers and diminished in deeper waters (Fig. 5C and Table S9). Notably, nearshore surface microbiomes, strongly shaped by terrestrial and coastal influences, exhibited enhanced horizontal connectivity compared to their counterparts in middle and bottom layers. Within these deeper layers, prokaryotes demonstrated substantially higher transfer capacity than microeukaryotes.

Regional differentiation further shaped microbial distribution. The SY contained more unique microeukaryotic taxa throughout the water column (Fig. S11). In contrast, the BH exhibited the highest vertical connectivity, attributable to its greater dispersal capacity. These findings demonstrate that horizontal gradients exert a stronger influence on coastal microbiome connectivity than vertical gradients, primarily driven by prokaryotic dispersal and modulated by variations in water depth and coastline proximity.

### Microbial dynamics and their causing environmental factors

**Fig 6:**
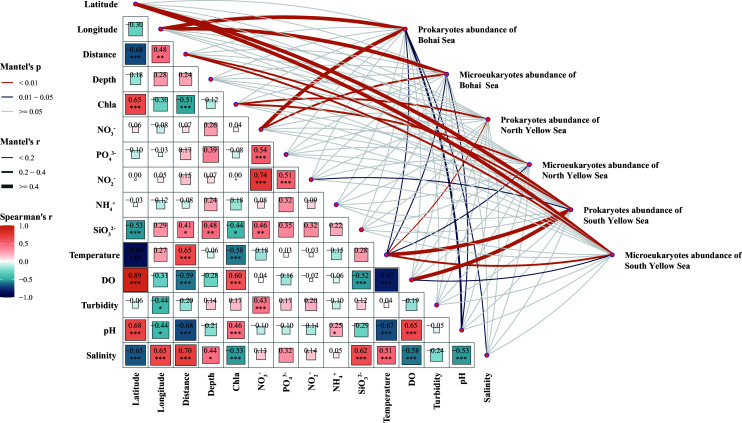
Correlations among environmental variables and microbial communities in the Bohai-Yellow Sea.

Microbial dynamics exhibited clear environmental correlations across the Bohai-Yellow Sea, with horizontal gradients exerting stronger influences than vertical stratification (Fig. 6; Fig. S4). Among the horizontal factors, distance to coastline showed significant positive correlations with salinity and temperature, but negative correlations with Chl a, dissolved oxygen (DO), and pH (|*r*| > 0.5, *p*.adj < 0.05). Similarly, geographic coordinates (latitude and longitude) were closely linked to variations in temperature, DO, pH, salinity, Chl a, and nutrient concentrations (|*r*| > 0.5, *p*.adj < 0.05). In contrast, depth was only correlated with the vertical distribution of salinity and silicate (|*r*| > 0.4, *p*.adj < 0.05).

Region-specific analyses further showed the primacy of horizontal drivers. In the BH both prokaryotic and microeukaryotic communities correlated significantly with multiple horizontal factors—including longitude, nitrate, temperature, DO, and pH—while prokaryotes additionally responded to salinity. In the NY, horizontal control remained evident: the variation of prokaryotic composition correlated solely with the temperature gradient, while microeukaryotes were linked to variations in ammonium and Chl a. Similarly, microbial dynamics in the SY were strongly associated with horizontal gradients such as distance to coastline, temperature, and DO, with prokaryotes further influenced by latitude (Fig. 6; Fig. S10). db-RDA analysis results showed that distance from coastline consistently exerted stronger independent effects than depth on both prokaryotic and microeukaryotic communities, with depth effects often non-significant (Table S7).

## DISCUSSION

### Horizontal gradients outweigh vertical stratification in coastal microbial assembly

Our study demonstrates that horizontal environmental gradients, particularly latitudinal transitions and distance to coastline, exert a stronger influence on microbial community structure than vertical stratification in the Bohai-Yellow Sea system. This finding aligns with large-scale patterns of latitudinal diversity gradients (9) but contrasts with highly stratified open-ocean systems where depth is the dominant organizing factor (48). The key role of distance to coastline in driving compositional turnover, especially among microeukaryotes, reinforces the importance of environmentally mediated distance–decay relationships in coastal oceans (5).

Niche breadth patterns further demonstrate this horizontal dominance. Prokaryotes showed pronounced southward niche expansion, whereas microeukaryotes responded more weakly, reflecting domain-specific ecological strategies. This extends global evidence of prokaryotic environmental adaptability by demonstrating its clear structuring along horizontal gradients (49). The limited niche variation along vertical gradients, except in the deeply stratified South Yellow Sea, reinforces that horizontal variability in salinity, nutrients, and terrestrial inputs exerts stronger selection than depth-related physicochemical changes (1, 18).

Mechanistically, stochastic processes dominated community assembly throughout the region. The latitudinal increase in dispersal limitation, particularly evident in the South Yellow Sea, indicates a reduction in effective microbial exchange along the horizontal environmental gradient. Within this framework, prokaryotes maintained consistently higher dispersal potential and broader niche breadth than microeukaryotes, enabling stronger horizontal connectivity—especially within intermediate water masses that may serve as dispersal corridors (30). In contrast, microeukaryotes exhibited constrained dispersal and narrower niches, resulting in higher regional endemicity. This fundamental difference in dispersal ecology, coupled with the transition from stable, modular networks in the semi-enclosed BH to vulnerable, interconnected structures in the open Yellow Sea, reveals how horizontal gradients shape ecosystem stability by modulating microbial interaction patterns.

### Horizontal gradients drive depth-dependent decline in microbial network robustness

Microbial network stability exhibited a depth-dependent decline along the Bohai–Yellow Sea with increasing environmental heterogeneity and water depth. The BH fostered highly modular, prokaryote-dominated networks, likely facilitated by substantial terrestrial inputs and restricted hydrographic exchange (50, 51). These networks were stabilized by key prokaryotic taxa, including NS2b marine group, *Candidatus* Aquiluna, *Porticoccus*, and SAR11 clade, via functional redundancy and high dispersal capacity (43, 52).

In contrast, the Yellow Sea networks transitioned toward microeukaryote-dominated architectures, with Sarcinochrysidales and Dinophyceae acting as network hubs. Both Sarcinochrysidales and Dinophyceae are mixotrophs coupling photosynthesis with phagotrophy, linking primary production to microbial loop dynamics via top-down control on prokaryotes and carbon transfer to higher trophic levels (23, 53, 54). Yet nutritional plasticity in these taxa entails physiological trade-offs that constrain growth to specific resource conditions (55). Large-scale biogeographic analyses confirm that mixotrophic dinoflagellates, despite being generalists, thrive optimally in tropical oligotrophic rather than temperate productive waters (56, 57). In the stratified Yellow Sea, such constraints restrict these microeukaryotes to narrower niches, increasing their vulnerability and explaining the greater community network fragility observed in deeper environments.

The variation in keystone taxa underscores the divergent ecological strategies that characterize each region’s microbial network. The SAR11 clade, for instance, served as a connector in the redundancy-rich BH and a module hub in the NY. The high abundance and metabolic versatility of SAR11 (58, 59) contribute directly to network persistence in coastal regions where it was a key taxon. SAR11 ecotypes exhibit clear biogeographic patterns across the oceans, with their relative abundances strongly correlated with factors like temperature and latitude (60). Furthermore, specific environmental pressures shape the functional composition of SAR11 communities, as evidenced by the increased abundance of nitrate reductase (nar)-carrying subclades under declining oxygen conditions in the Bay of Bengal (61). Its absence as a keystone species in the SY likely reflects niche partitioning under intensified environmental filtering (62, 63). Thus, the loss of prokaryotic generalists such as SAR11 along the depth gradient contributed directly to the increasing fragility of microbial networks toward the open sea.

### A dominant horizontal mode of microbial connectivity in coastal oceans

Horizontal gradients emerged as the principal driver of microbial connectivity and regional assembly, outweighing the influence of vertical gradients in this coastal system. The strong microbial abundance-environment correlations with salinity, temperature, and Chl a reflect steep coastal-offshore environmental gradients that amplify the influence of horizontal transport on community turnover. Horizontal connectivity varied substantially with latitude and coastal distance, whereas vertical connectivity remained relatively stable. This pattern reflects strong horizontal environmental filtering that restricts microbial exchange, consistent with the dispersal limitation observed in community assembly.

This dominance of horizontal processes establishes a distinct pattern from the open ocean, where sinking particulate organic matter sustains strong vertical microbial connectivity from surface to mesopelagic depths (30, 48, 64). Vertical connectivity in the shallow Bohai–Yellow Sea system attenuated with increasing stratification from the well-mixed BH to the stratified SY, consistent with water column stability acting as a barrier to vertical mixing (1, 65). The higher vertical connectivity of prokaryotes relative to microeukaryotes further reflects their broader environmental tolerance and dispersal potential (66, 67).

Overall, our research establishes that microbial connectivity in shallow, land-influenced, and human-affected coastal oceans such as the Bohai and Yellow Seas is driven primarily by horizontal gradients. We propose that the Bohai–Yellow Sea functions as a large metacommunity whose biogeography is mainly shaped by horizontal transport and environmental gradients. Although constrained to a single spring cruise, our findings established a baseline for future multi-seasonal investigations into the persistence of this horizontal connectivity mode and its potential seasonal variability. This conceptual advance highlights a dominant horizontal mode of connectivity, underscoring the need for future modeling frameworks to incorporate horizontal advection and regional dispersal as key predictors of microbial responses to anthropogenic change and climate-driven perturbations.

### Conclusion

This study establishes horizontal environmental gradients, primarily driven by distance to coastline and latitude, as the principal factor shaping microbial community structure, connectivity, and stability in the Bohai-Yellow Sea. Overwhelming the influence of vertical stratification, these gradients favored prokaryotes with stronger dispersal capacity and broader niches, especially in intermediate waters. The resulting latitudinal transition of microbiomes led to a marked decline in community network stability, shifting networks from robust, prokaryote dominated systems in the Bohai Sea to vulnerable, microeukaryote regulated architectures in the Yellow Sea. These findings establish a mechanistic framework based on horizontal connectivity that enables the prediction of microbial responses to anthropogenic and climate change in coastal oceans.

## Data Availability

The raw sequence data reported in this paper have been deposited in the Genome Sequence Archive of the National Genomics Data Center, China National Center for Bioinformation / Beijing Institute of Genomics, Chinese Academy of Sciences, and are publicly accessible at https://ngdc.cncb.ac.cn/gsa under accessions CRA033419 and CRA033420.

## SUPPLEMENTAL MATERIAL

Supplemental material aem.00448-26-s0001.docx Fig. S1 to S11; Tables S1 to S9.

ASM does not own the copyrights to Supplemental Material that may be linked to, or accessed through, an article. The authors have granted ASM a non-exclusive, world-wide license to publish the Supplemental Material files. Please contact the corresponding author directly for reuse.

## References

[1] Lima-Mendez G, Faust K, Henry N, Decelle J, Colin S, Carcillo F, Chaffron S, Ignacio-Espinosa JC, Roux S, Vincent F, et al.. 2015. Ocean plankton. determinants of community structure in the global plankton interactome. Science 348:1262073. doi:10.1126/science.1262073 25999517

[2] Azam F, Malfatti F. 2007. Microbial structuring of marine ecosystems. Nat Rev Microbiol 5:782–791. doi:10.1038/nrmicro1747 17853906

[3] Falkowski PG, Fenchel T, Delong EF. 2008. The microbial engines that drive earth’s biogeochemical cycles. Science 320:1034–1039. doi:10.1126/science.1153213 18497287

[4] Field CB, Behrenfeld MJ, Randerson JT, Falkowski P. 1998. Primary production of the biosphere: integrating terrestrial and oceanic components. Science 281:237–240. doi:10.1126/science.281.5374.237 9657713

[5] Hanson CA, Fuhrman JA, Horner-Devine MC, Martiny JBH. 2012. Beyond biogeographic patterns: processes shaping the microbial landscape. Nat Rev Microbiol 10:497–506. doi:10.1038/nrmicro2795 22580365

[6] Salazar G, Paoli L, Alberti A, Huerta-Cepas J, Ruscheweyh H-J, Cuenca M, Field CM, Coelho LP, Cruaud C, Engelen S, et al.. 2019. Gene expression changes and community turnover differentially shape the global ocean metatranscriptome. Cell 179:1068–1083. doi:10.1016/j.cell.2019.10.014 31730850 PMC6912165

[7] Lian K, Liu F, Li Y, Wang C, Zhang C, McMinn A, Wang M, Wang H. 2023. Environmental gradients shape microbiome assembly and stability in the East China sea. Environ Res 238:117197. doi:10.1016/j.envres.2023.117197 37783325

[8] Zinger L, Amaral-Zettler LA, Fuhrman JA, Horner-Devine MC, Huse SM, Welch DBM, Martiny JBH, Sogin M, Boetius A, Ramette A. 2011. Global patterns of bacterial beta-diversity in seafloor and seawater ecosystems. PLoS One 6:e24570. doi:10.1371/journal.pone.0024570 21931760 PMC3169623

[9] Sul WJ, Oliver TA, Ducklow HW, Amaral-Zettler LA, Sogin ML. 2013. Marine bacteria exhibit a bipolar distribution. Proc Natl Acad Sci USA 110:2342–2347. doi:10.1073/pnas.1212424110 23324742 PMC3568360

[10] Martiny JBH, Bohannan BJM, Brown JH, Colwell RK, Fuhrman JA, Green JL, Horner-Devine MC, Kane M, Krumins JA, Kuske CR, et al.. 2006. Microbial biogeography: putting microorganisms on the map. Nat Rev Microbiol 4:102–112. doi:10.1038/nrmicro1341 16415926

[11] Hutchins DA, Fu F. 2017. Microorganisms and ocean global change. Nat Microbiol 2:17058. doi:10.1038/nmicrobiol.2017.58 28540925

[12] Papke RT, Ramsing NB, Bateson MM, Ward DM. 2003. Geographical isolation in hot spring cyanobacteria. Environ Microbiol 5:650–659. doi:10.1046/j.1462-2920.2003.00460.x 12871232

[13] Whitaker RJ, Grogan DW, Taylor JW. 2003. Geographic barriers isolate endemic populations of hyperthermophilic archaea. Science 301:976–978. doi:10.1126/science.1086909 12881573

[14] Cloern JE. 2001. Our evolving conceptual model of the coastal eutrophication problem. Mar Ecol Prog Ser 210:223–253. doi:10.3354/meps210223

[15] Niu T, Xu Y, Chen J, Qin L, Li Z, Yang Y, Liang J. 2023. Bacterial taxonomic and functional profiles from Bohai Sea to northern Yellow Sea. Front Microbiol 14:1139950. doi:10.3389/fmicb.2023.1139950 36910186 PMC9995391

[16] Levin LA, Liu K-K, Emeis K-C, Breitburg DL, Cloern J, Deutsch C, Giani M, Goffart A, Hofmann EE, Lachkar Z, et al.. 2015. Comparative biogeochemistry–ecosystem–human interactions on dynamic continental margins. J Mar Syst 141:3–17. doi:10.1016/j.jmarsys.2014.04.016

[17] Herlemann DPR, Labrenz M, Jürgens K, Bertilsson S, Waniek JJ, Andersson AF. 2011. Transitions in bacterial communities along the 2000 km salinity gradient of the Baltic Sea. ISME J 5:1571–1579. doi:10.1038/ismej.2011.41 21472016 PMC3176514

[18] Wang H, Chen F, Zhang C, Wang M, Kan J. 2021. Estuarine gradients dictate spatiotemporal variations of microbiome networks in the Chesapeake Bay. Environ Microbiome 16:22. doi:10.1186/s40793-021-00392-z 34838139 PMC8627074

[19] Wang Z, Juarez DL, Pan J-F, Blinebry SK, Gronniger J, Clark JS, Johnson ZI, Hunt DE. 2019. Microbial communities across nearshore to offshore coastal transects are primarily shaped by distance and temperature. Environ Microbiol 21:3862–3872. doi:10.1111/1462-2920.14734 31286605

[20] Ohore OE, Ifon BE, Wang Y, Kazmi S, Zhang J, Sanganyado E, Jiao X, Liu W, Wang Z. 2023. Vertical changes in water depth and environmental variables drove the antibiotics and antibiotic resistomes distribution, and microbial food web structures in the estuary and marine ecosystems. Environ Int 178:108118. doi:10.1016/j.envint.2023.108118 37517178

[21] Ohore OE, Wei Y, Wang J, Wang Y, Ifon BE, Liu W, Wang Z. 2022. Vertical characterisation of phylogenetic divergence of microbial community structures, interaction, and sustainability in estuary and marine ecosystems. Sci Total Environ 851:158369. doi:10.1016/j.scitotenv.2022.158369 36049676

[22] Xian W-D, Ding J, Chen J, Qu W, Cao P, Tang C, Liu X, Zhang Y, Li J-L, Wang P, et al.. 2024. Distinct assembly processes structure planktonic bacterial communities among near- and offshore ecosystems in the yangtze river estuary. Microb Ecol 87:42. doi:10.1007/s00248-024-02350-x 38356037 PMC11385042

[23] Xu S, Liu Y, Zhang Z, Xu Y, Qi Z. 2022. Distributional pattern of bacteria, protists, and diatoms in ocean according to water depth in the northern south China Sea. Microbiol Spectr 10:e0275921. doi:10.1128/spectrum.02759-21 36222702 PMC9769685

[24] Halpern BS, Walbridge S, Selkoe KA, Kappel CV, Micheli F, D’Agrosa C, Bruno JF, Casey KS, Ebert C, Fox HE, et al.. 2008. A global map of human impact on marine ecosystems. Science 319:948–952. doi:10.1126/science.1149345 18276889

[25] Liang S, Li S, Guo J, Yang Y, Xu Z, Zhang M, Li H, Yu X, Ma H, Wang X. 2023. Source, composition, and reactivity of particulate organic matter along the Changjiang Estuary salinity gradient and adjacent sea. Mar Chem 252:104245. doi:10.1016/j.marchem.2023.104245

[26] Li X, Chen H, Jiang X, Yu Z, Yao Q. 2017. Impacts of human activities on nutrient transport in the Yellow River: the role of the water-sediment regulation scheme. Science of The Total Environment 592:161–170. doi:10.1016/j.scitotenv.2017.03.098 28319703

[27] Liu SM. 2015. Response of nutrient transports to water–sediment regulation events in the Huanghe basin and its impact on the biogeochemistry of the Bohai. J Mar Syst 141:59–70. doi:10.1016/j.jmarsys.2014.08.008

[28] Zhang S, Li Q, Zou Y, Liu B, Yang J, Zheng H, Liu G. 2024. Using isotopic lead and strontium in sediments to trace natural and anthropogenic sources in the Bohai Sea. Sci Rep 14:30267. doi:10.1038/s41598-024-81493-w 39632994 PMC11618753

[29] Fang Y, Chen Y, Tian C, Wang X, Lin T, Hu L, Li J, Zhang G, Luo Y. 2018. Cycling and budgets of organic and black carbon in coastal Bohai Sea, China: impacts of natural and anthropogenic perturbations. Global Biogeochem Cycles 32:971–986. doi:10.1029/2017GB005863

[30] Chen S, Xie Z-X, Yan K-Q, Chen J-W, Li D-X, Wu P-F, Peng L, Lin L, Dong C-M, Zhao Z, et al.. 2024. Functional vertical connectivity of microbial communities in the ocean. Sci Adv 10:eadj8184. doi:10.1126/sciadv.adj8184 38781332 PMC11114224

[31] Luo M, Zhu J, Jia J, Zhang H, Zhao J. 2024. Progress on network modeling and analysis of gut microecology: a review. Appl Environ Microbiol Appl Environ Microbiol 90:e0009224. doi:10.1128/aem.00092-24 PMC11207142 38415584

[32] Srinivasan S, Jnana A, Murali TS. 2024. Modeling microbial community networks: methods and tools for studying microbial interactions. Microb Ecol 87:56. doi:10.1007/s00248-024-02370-7 38587642 PMC11001700

[33] Xiao N, Zhou A, Kempher ML, Zhou BY, Shi ZJ, Yuan M, Guo X, Wu L, Ning D, Van Nostrand J, et al.. 2022. Disentangling direct from indirect relationships in association networks. Proc Natl Acad Sci USA 119:e2109995119. doi:10.1073/pnas.2109995119 34992138 PMC8764688

[34] Liu Z, Wei H, Lozovatsky ID, Fernando HJS. 2009. Late summer stratification, internal waves, and turbulence in the Yellow Sea. J Mar Syst 77:459–472. doi:10.1016/j.jmarsys.2008.11.001

[35] Cheung MK, Au CH, Chu KH, Kwan HS, Wong CK. 2010. Composition and genetic diversity of picoeukaryotes in subtropical coastal waters as revealed by 454 pyrosequencing. ISME J 4:1053–1059. doi:10.1038/ismej.2010.26 20336159

[36] Apprill A, McNally S, Parsons R, Weber L. 2015. Minor revision to V4 region SSU rRNA 806R gene primer greatly increases detection of SAR11 bacterioplankton. Aquat Microb Ecol 75:129–137. doi:10.3354/ame01753

[37] Parada AE, Needham DM, Fuhrman JA. 2016. Every base matters: assessing small subunit rRNA primers for marine microbiomes with mock communities, time series and global field samples. Environ Microbiol 18:1403–1414. doi:10.1111/1462-2920.13023 26271760

[38] Wessel P, Smith WHF. 1996. A global, self‐consistent, hierarchical, high‐resolution shoreline database. J Geophys Res 101:8741–8743. doi:10.1029/96JB00104

[39] Sloan WT, Lunn M, Woodcock S, Head IM, Nee S, Curtis TP. 2006. Quantifying the roles of immigration and chance in shaping prokaryote community structure. Environ Microbiol 8:732–740. doi:10.1111/j.1462-2920.2005.00956.x 16584484

[40] Webb CO, Ackerly DD, Kembel SW. 2008. Phylocom: software for the analysis of phylogenetic community structure and trait evolution. Bioinformatics 24:2098–2100. doi:10.1093/bioinformatics/btn358 18678590

[41] Arnault G, Mony C, Vandenkoornhuyse P. 2023. Plant microbiota dysbiosis and the Anna Karenina Principle. Trends Plant Sci 28:18–30. doi:10.1016/j.tplants.2022.08.012 36127241

[42] Jiao S, Chu H, Zhang B, Wei X, Chen W, Wei G. 2022. Linking soil fungi to bacterial community assembly in arid ecosystems. iMeta 1:iMeta doi:10.1002/imt2.2 PMC10989902 38867731

[43] Jiao S, Lu Y. 2020. Abundant fungi adapt to broader environmental gradients than rare fungi in agricultural fields. Glob Chang Biol 26:4506–4520. doi:10.1111/gcb.15130 32324306

[44] Deng Y, Jiang Y-H, Yang Y, He Z, Luo F, Zhou J. 2012. Molecular ecological network analyses. BMC Bioinformatics 13:113. doi:10.1186/1471-2105-13-113 22646978 PMC3428680

[45] Zhou J, Deng Y, Luo F, He Z, Tu Q, Zhi X. 2010. Functional molecular ecological networks. mBio 1:e00169–10. doi:10.1128/mBio.00169-10 20941329 PMC2953006

[46] Zhou J, Deng Y, Luo F, He Z, Yang Y. 2011. Phylogenetic molecular ecological network of soil microbial communities in response to elevated CO2. mBio 2:e00122-11. doi:10.1128/mBio.00122-11 21791581 PMC3143843

[47] Wan W, Gadd GM, Yang Y, Yuan W, Gu J, Ye L, Liu W. 2021. Environmental adaptation is stronger for abundant rather than rare microorganisms in wetland soils from the Qinghai-Tibet Plateau. Mol Ecol 30:2390–2403. doi:10.1111/mec.15882 33714213

[48] Mestre M, Ruiz-González C, Logares R, Duarte CM, Gasol JM, Sala MM. 2018. Sinking particles promote vertical connectivity in the ocean microbiome. Proc Natl Acad Sci USA 115:E6799–E6807. doi:10.1073/pnas.1802470115 29967136 PMC6055141

[49] Bahram M, Hildebrand F, Forslund SK, Anderson JL, Soudzilovskaia NA, Bodegom PM, Bengtsson-Palme J, Anslan S, Coelho LP, Harend H, et al.. 2018. Structure and function of the global topsoil microbiome. Nature 560:233–237. doi:10.1038/s41586-018-0386-6 30069051

[50] Wang Y, Zhou M, Yue X, Chen Y, Su D, Liu Z. 2025. Noctiluca scintillans bloom reshapes microbial community structure, interaction networks, and metabolism patterns in qinhuangdao coastal waters, China. Microorganisms 13:1959. doi:10.3390/microorganisms13081959 40871463 PMC12388393

[51] Zhao Y, Zhang T, Qi D, Xie L, Ni S-Q. 2024. Neglected dissimilatory nitrate reduction to ammonium pathway of nitrate reduction in the chinese marginal sea and its microbial community assembly driven by stochastic processes. ACS EST Water 4:1579–1589. doi:10.1021/acsestwater.3c00674

[52] Fuhrman JA, Cram JA, Needham DM. 2015. Marine microbial community dynamics and their ecological interpretation. Nat Rev Microbiol 13:133–146. doi:10.1038/nrmicro3417 25659323

[53] Stoecker DK, Hansen PJ, Caron DA, Mitra A. 2017. Mixotrophy in the marine plankton. Annu Rev Mar Sci 9:311–335. doi:10.1146/annurev-marine-010816-060617 27483121

[54] Jeong HJ, Yoo YD, Kim JS, Seong KA, Kang NS, Kim TH. 2010. Growth, feeding and ecological roles of the mixotrophic and heterotrophic dinoflagellates in marine planktonic food webs. Ocean Sci J 45:65–91. doi:10.1007/s12601-010-0007-2

[55] Edwards KF. 2019. Mixotrophy in nanoflagellates across environmental gradients in the ocean. Proc Natl Acad Sci USA 116:6211–6220. doi:10.1073/pnas.1814860116 30760589 PMC6442547

[56] Dong K, Wang Y, Zhang W, Li Q. 2024. Prevalence and preferred niche of small eukaryotes with mixotrophic potentials in the global ocean. Microorganisms 12:750. doi:10.3390/microorganisms12040750 38674694 PMC11051772

[57] Rihm G, Benedetti F, Bittner L. 2025. Do trophic strategies shape biogeography and environmental niches? Marine dinoflagellates as a case study. ISME Commun 5:ycaf153. doi:10.1093/ismeco/ycaf153 40989910 PMC12452278

[58] Fahimipour AK, Gross T. 2020. Mapping the bacterial metabolic niche space. Nat Commun 11:4887. doi:10.1038/s41467-020-18695-z 32985497 PMC7522980

[59] Morris JJ, Lenski RE, Zinser ER. 2012. The black queen hypothesis: evolution of dependencies through adaptive gene loss. mBio 3:e00036-12. doi:10.1128/mBio.00036-12 22448042 PMC3315703

[60] Brown MV, Lauro FM, DeMaere MZ, Muir L, Wilkins D, Thomas T, Riddle MJ, Fuhrman JA, Andrews‐Pfannkoch C, Hoffman JM, et al.. 2012. Global biogeography of SAR11 marine bacteria. Mol Syst Biol 8:595. doi:10.1038/msb.2012.28 22806143 PMC3421443

[61] Gu B, Liu J, Cheung S, Ho NHE, Tan Y, Xia X. 2022. Insights into prokaryotic community and its potential functions in nitrogen metabolism in the Bay of Bengal, a pronounced oxygen minimum zone. Microbiol Spectr 10. doi:10.1128/spectrum.00892-21 PMC9241787 35579458

[62] Chen Y-J, Leung PM, Wood JL, Bay SK, Hugenholtz P, Kessler AJ, Shelley G, Waite DW, Franks AE, Cook PLM, et al.. 2021. Metabolic flexibility allows bacterial habitat generalists to become dominant in a frequently disturbed ecosystem. ISME J 15:2986–3004. doi:10.1038/s41396-021-00988-w 33941890 PMC8443593

[63] Giovannoni SJ. 2017. SAR11 bacteria: the most abundant plankton in the oceans. Annu Rev Mar Sci 9:231–255. doi:10.1146/annurev-marine-010814-015934 27687974

[64] Liu S, Hu R, Strong PJ, Saleem M, Zhou Z, Luo Z, Wu Y, He Z, Wang C. 2023. Vertical connectivity of microbiome and metabolome reveals depth-dependent variations across a deep cold-seep water column. Environ Res 239:117310. doi:10.1016/j.envres.2023.117310 37805181

[65] Sunagawa S, Coelho LP, Chaffron S, Kultima JR, Labadie K, Salazar G, Djahanschiri B, Zeller G, Mende DR, Alberti A, et al.. 2015. Ocean plankton. Structure and function of the global ocean microbiome. Science 348:1261359. doi:10.1126/science.1261359 25999513

[66] Logares R, Deutschmann IM, Junger PC, Giner CR, Krabberød AK, Schmidt TSB, Rubinat-Ripoll L, Mestre M, Salazar G, Ruiz-González C, et al.. 2020. Disentangling the mechanisms shaping the surface ocean microbiota. Microbiome 8:55. doi:10.1186/s40168-020-00827-8 32312331 PMC7171866

[67] Zhang C, Liu F, Zou Y, Wang C, Zhang H, Wang B, Kan J, McMinn A, Wang H, Wang M. 2024. Vertical heterogeneity enhances network complexity and stability of co-occurrence microbes in the eastern Indian Ocean. Environ Res 263:120225. doi:10.1016/j.envres.2024.120225 39448009

