# Supplementary material for: A horizontal connectivity mode in coastal oceans: transport overrides stratification to govern microbiome network stability
Source: Appl Environ Microbiol. 2026 May 27;92(6):e00448-26. doi: 10.1128/aem.00448-26 (PMC13274344; doi:10.1128/aem.00448-26)
Supplement: Supplemental material — Fig. S1 to S11; Tables S1 to S9. [file aem.00448-26-s0001.docx]

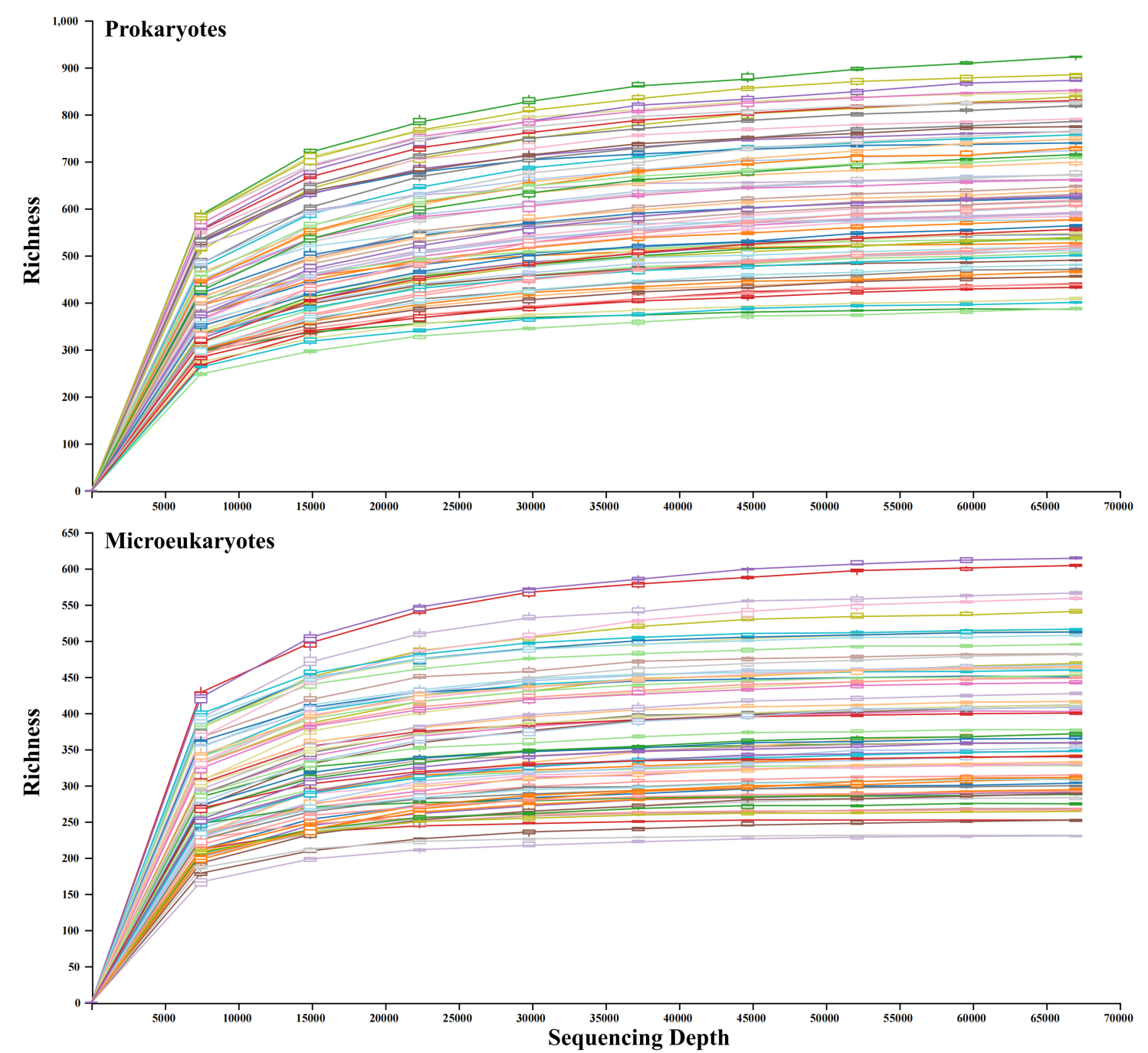


**FIG S1** Alpha rarefaction curve for the ASV numbers of 16S/18S rDNA sequenced.


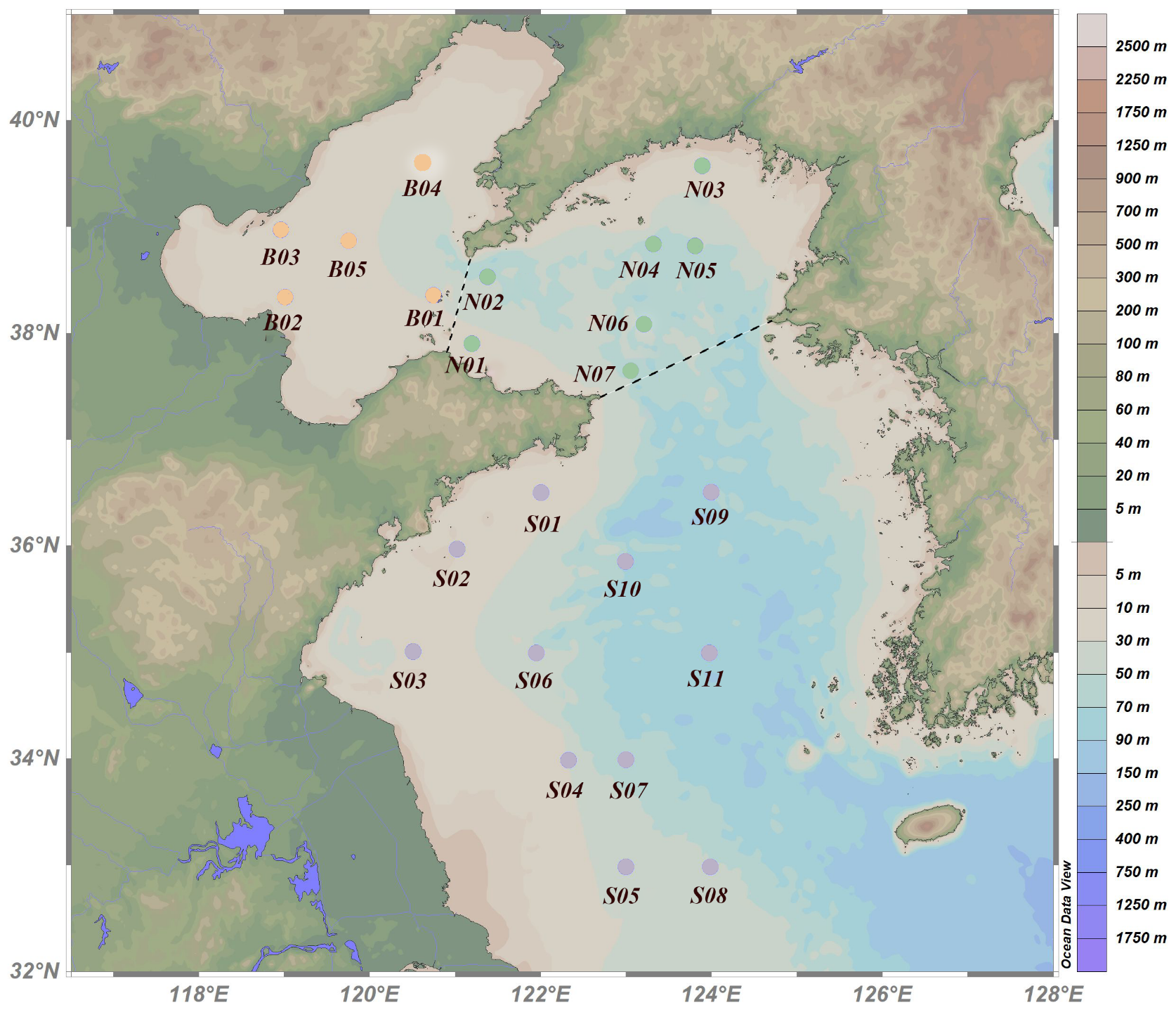


**FIG S2** Sampling stations in the Bohai-Yellow Sea.


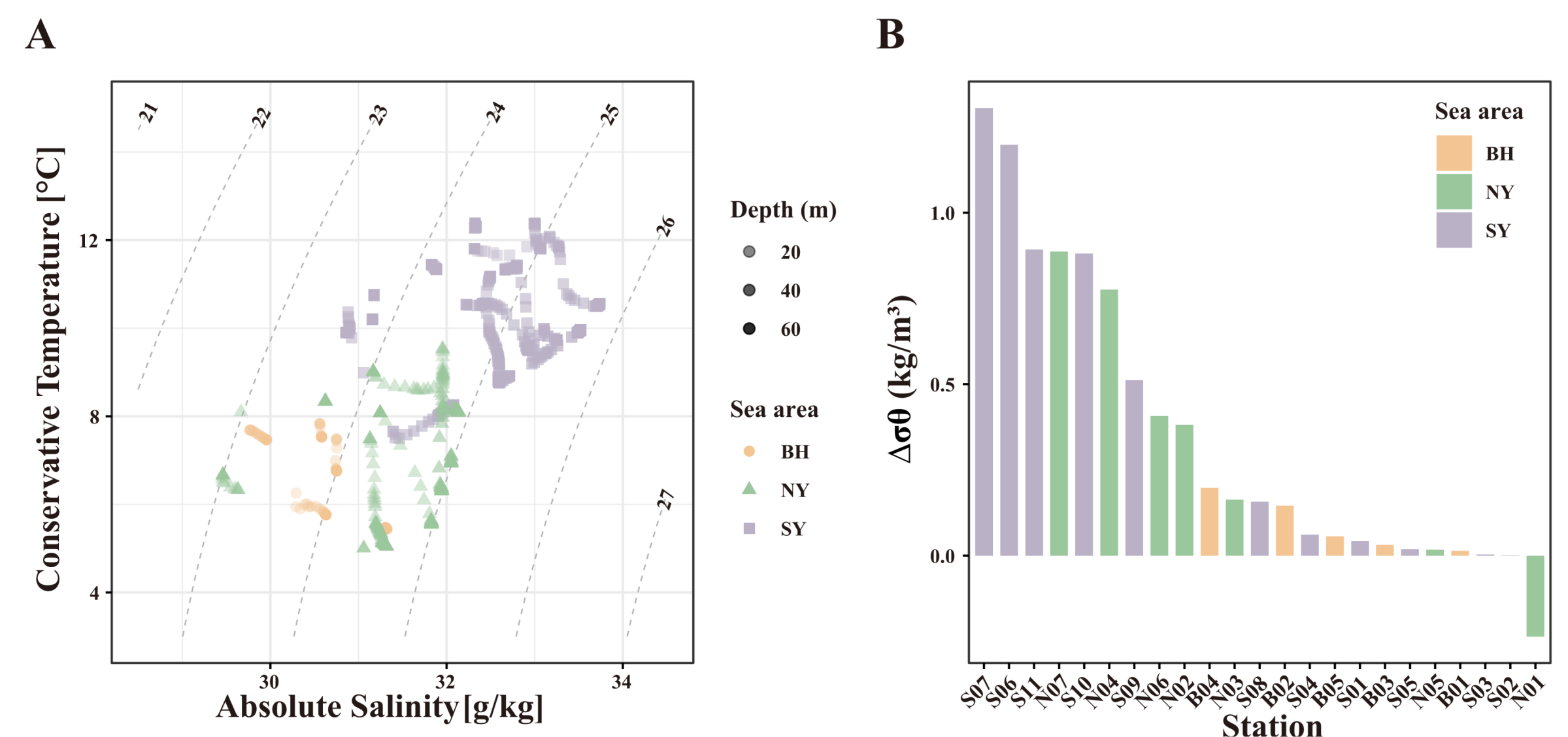


**FIG S3** Hydrological characteristics in the Bohai-Yellow Sea. (A) Temperature–Salinity (T-S) diagram with dashed lines indicating isopycnals (potential density anomaly σ₀, kg/m³). (B) The variation of vertical density between surface and bottom waters at each station.


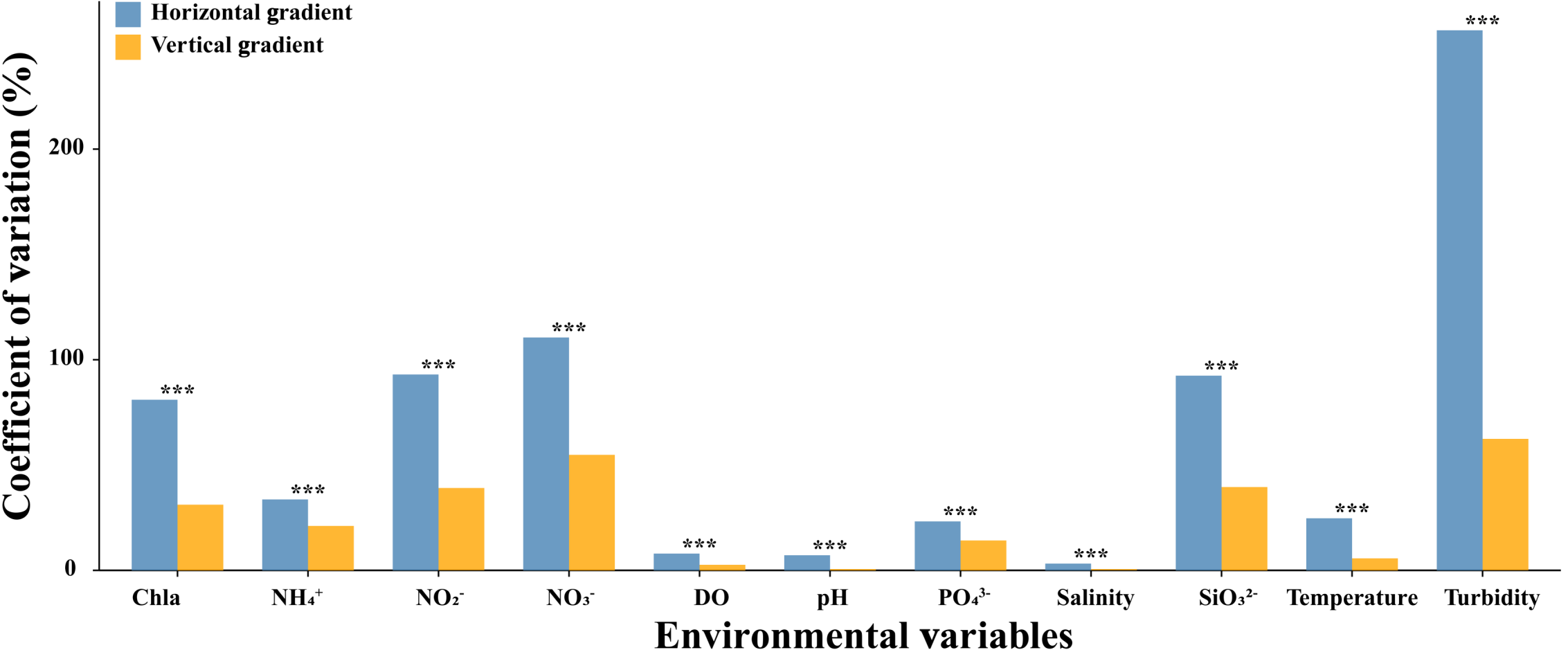


**FIG S4** Comparison of the coefficient of variation (CV) of environmental variables between horizontal and vertical gradients.


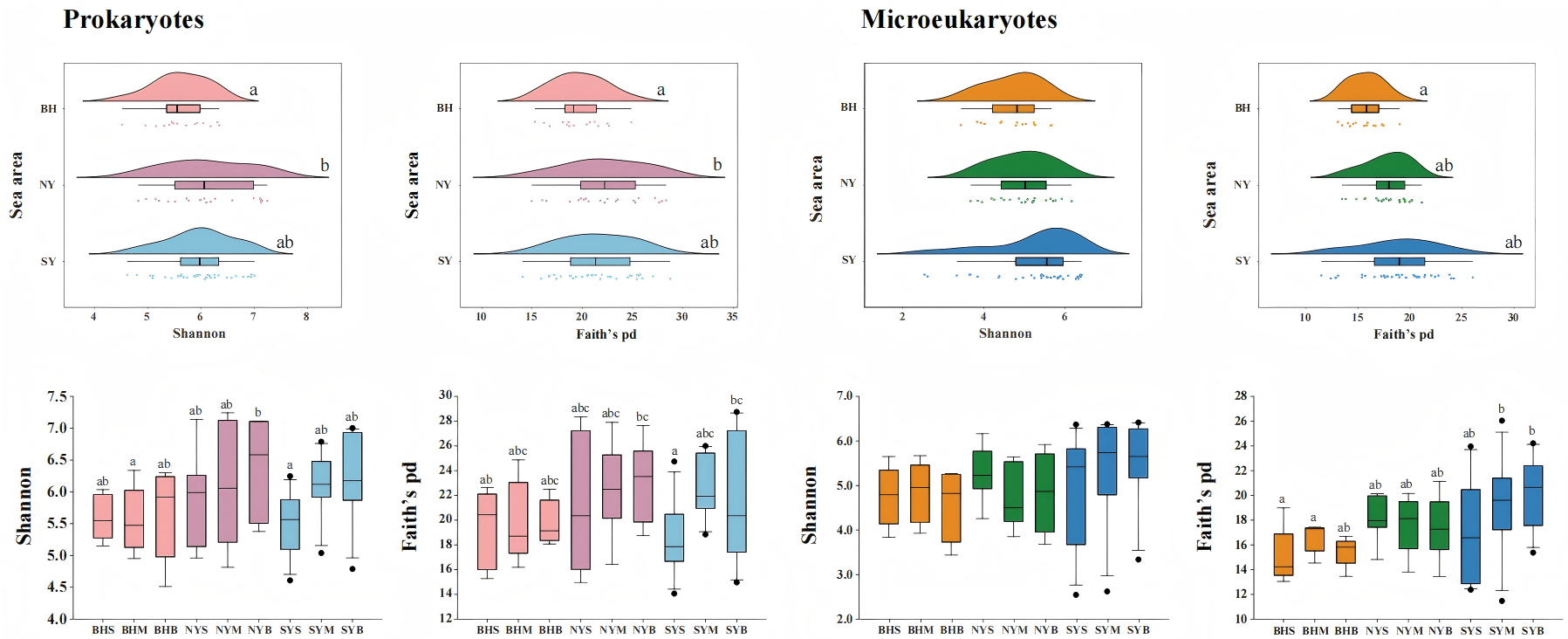


**FIG S5** Microbial community diversity in the Bohai–Yellow Sea. (A) Shannon index and Faith's PD along horizontal and vertical gradients. (B) Spatial distribution of species richness. BHS/BHM/BHB: Bohai Sea surface/middle/bottom; NYS/NYM/NYB: North Yellow Sea surface/middle/bottom; SYS/SYM/SYB: South Yellow Sea surface/middle/bottom.


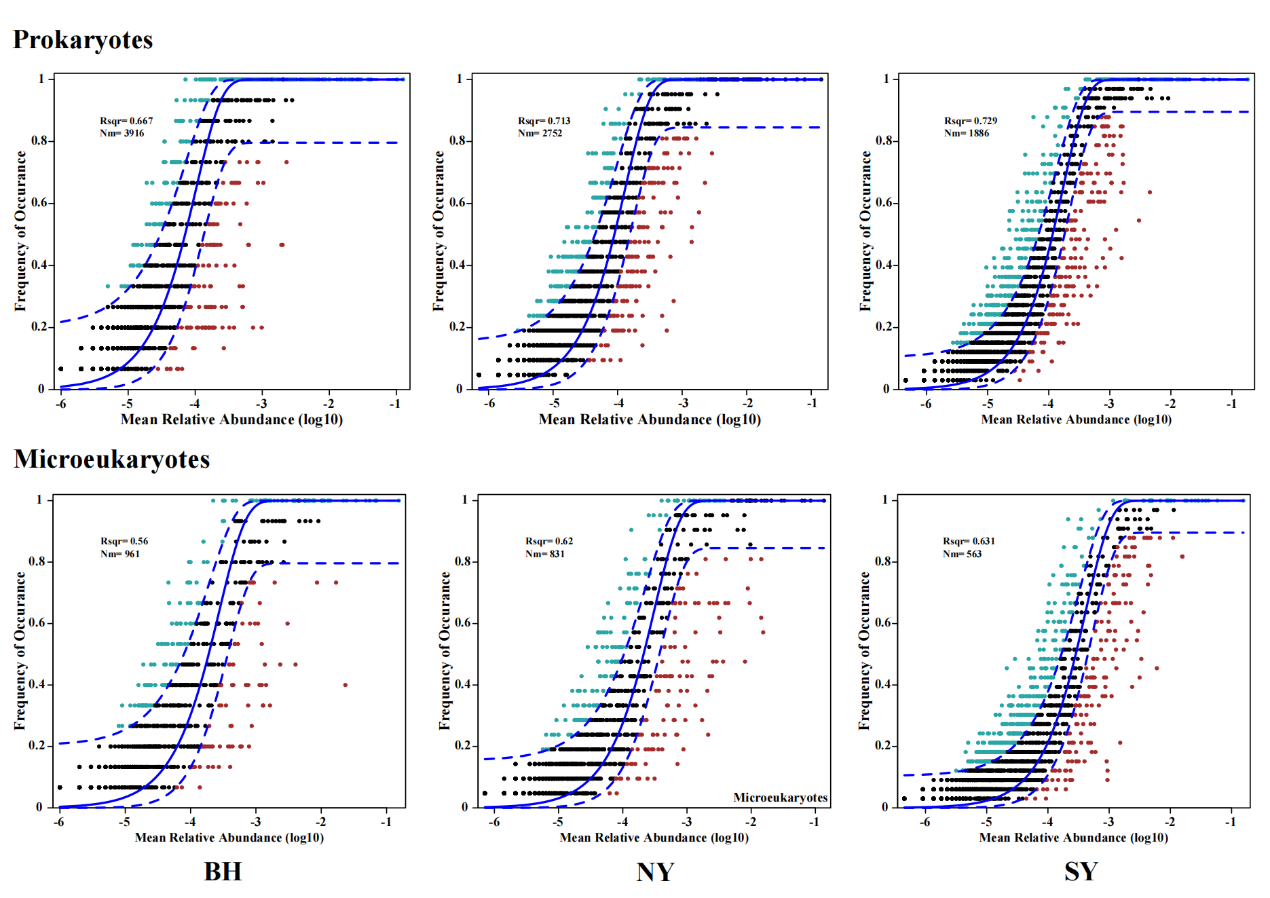


**FIG S6** Neutral community model fit for prokaryotic and eukaryotic microbiomes in the Bohai-Yellow Sea. Solid lines denote the optimal model fit, whereas dashed lines represent 95% confidence intervals of predicted values.


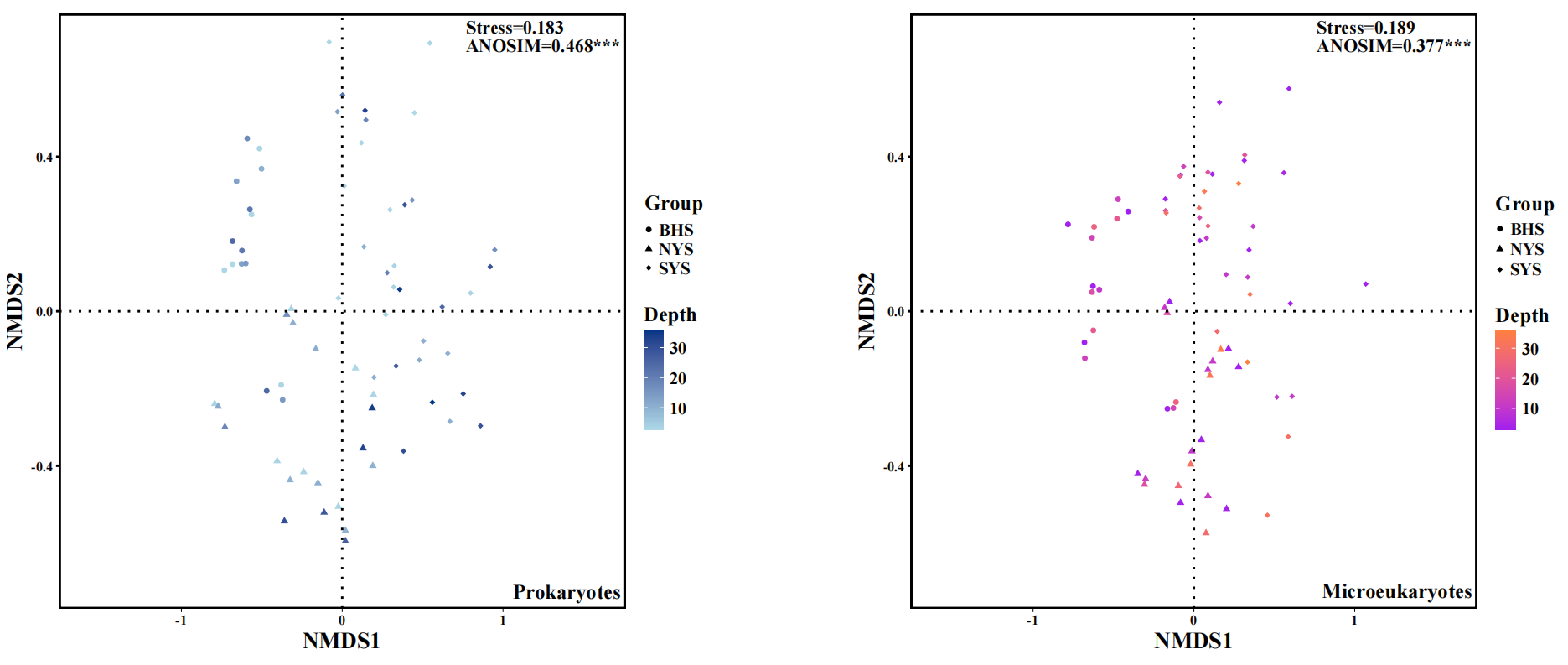


**FIG S7** Non-metric multidimensional scaling (NMDS) ordination of seawater microbial communities based on Bray-Curtis distance along vertical gradients.(BHS: Bohai Sea; NYS: North Yellow Sea. SYS: South Yellow Sea.)


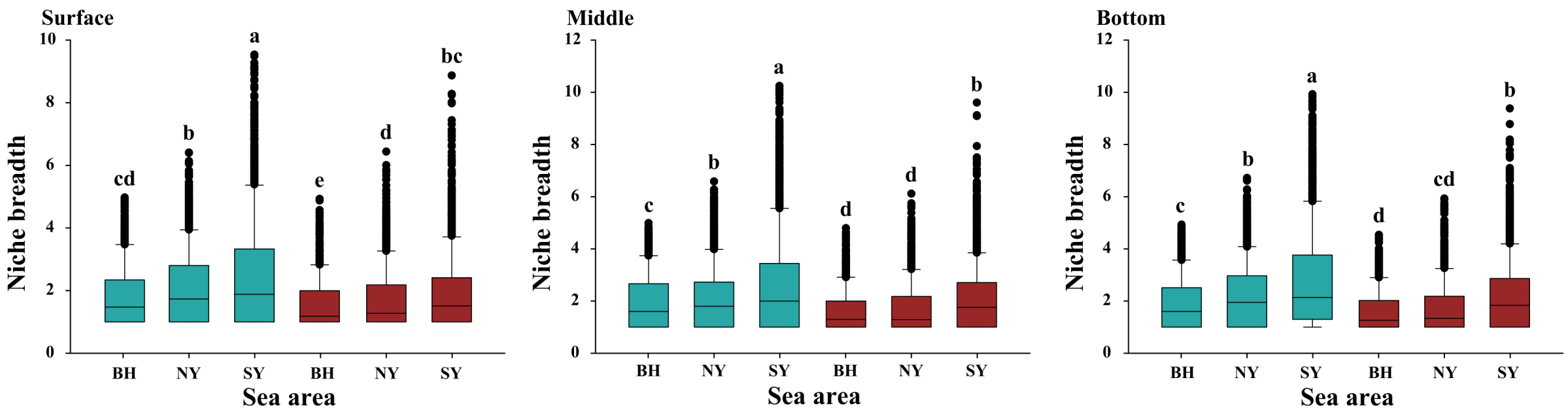


**FIG S8** Niche breadth along vertical gradients of the Bohai-Yellow Sea.


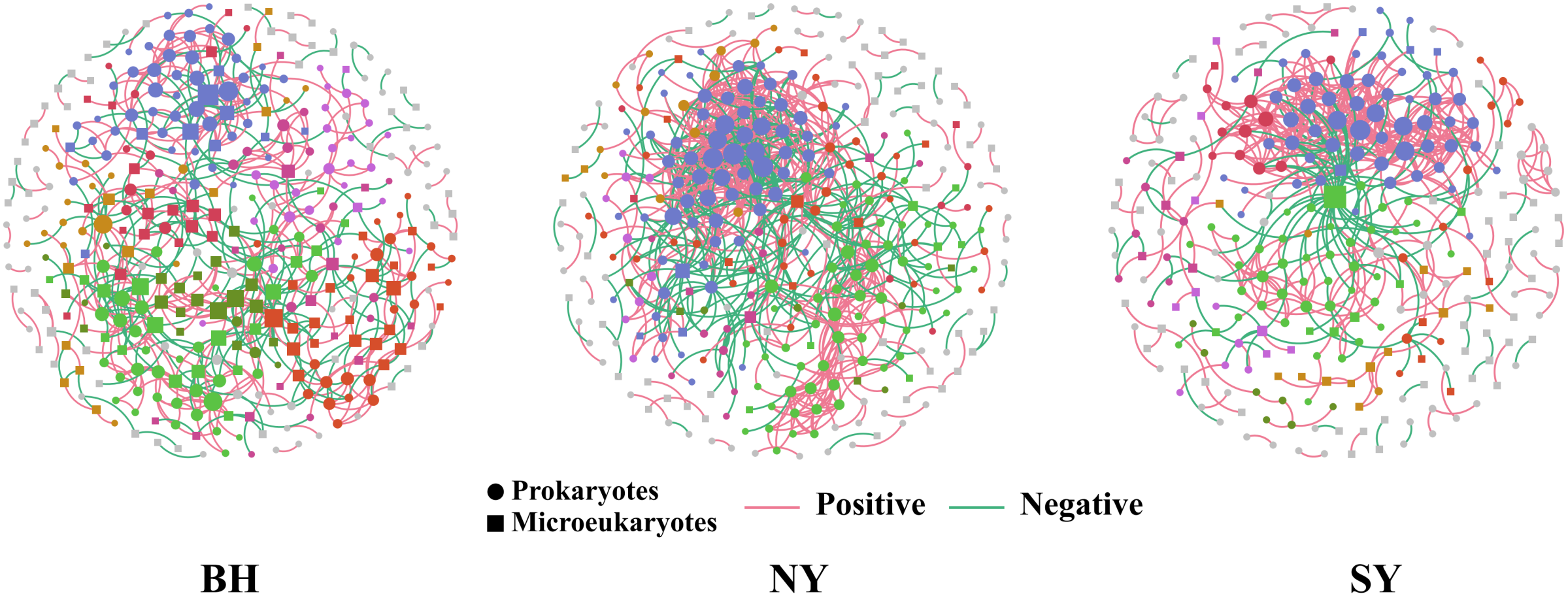


**FIG S9** Co-occurrence network patterns of microbial communities in the Bohai-Yellow Sea.


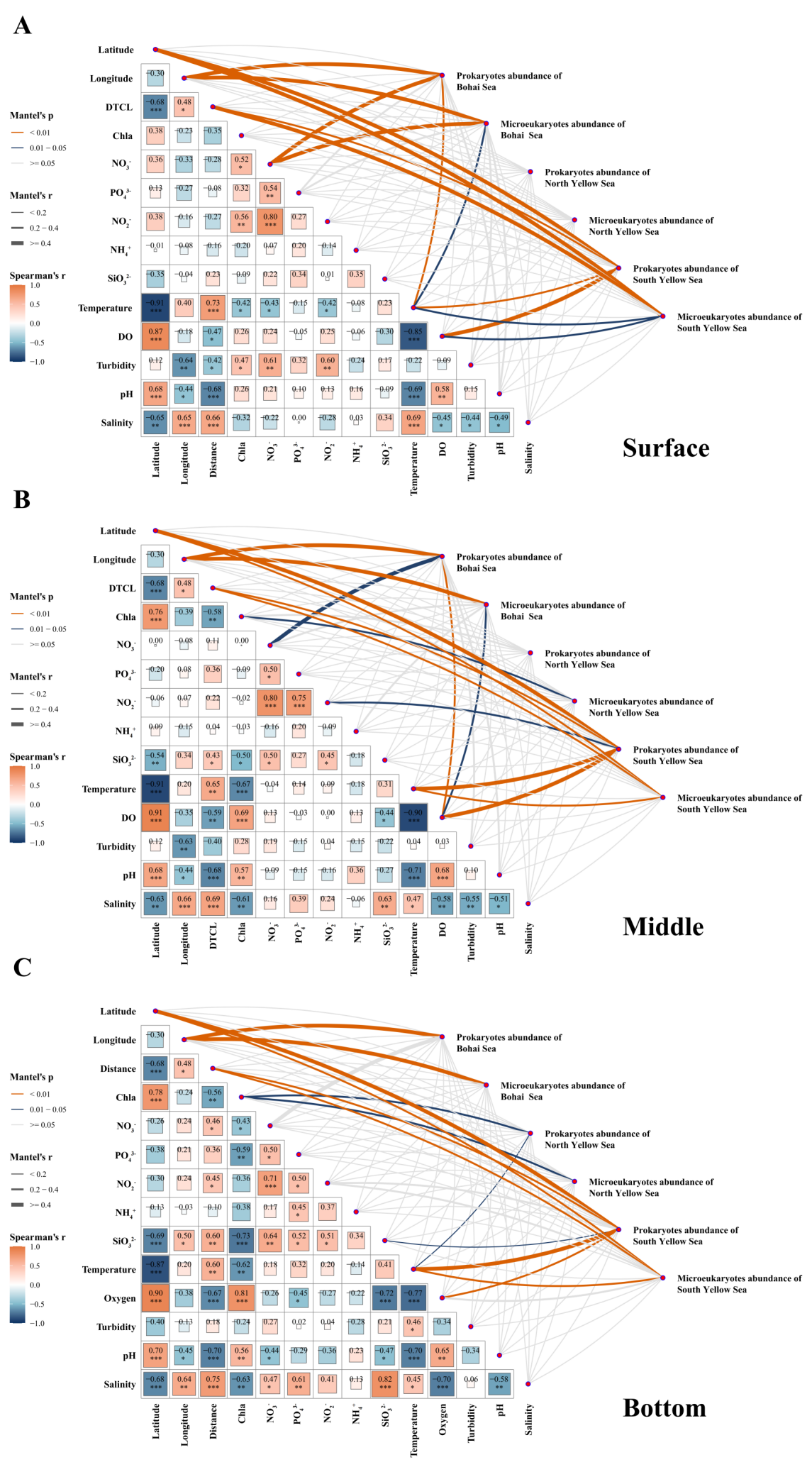


**FIG S10** Relations among environmental factors and microbial communities in the surface (A), middle (B), and bottom (C) layers.


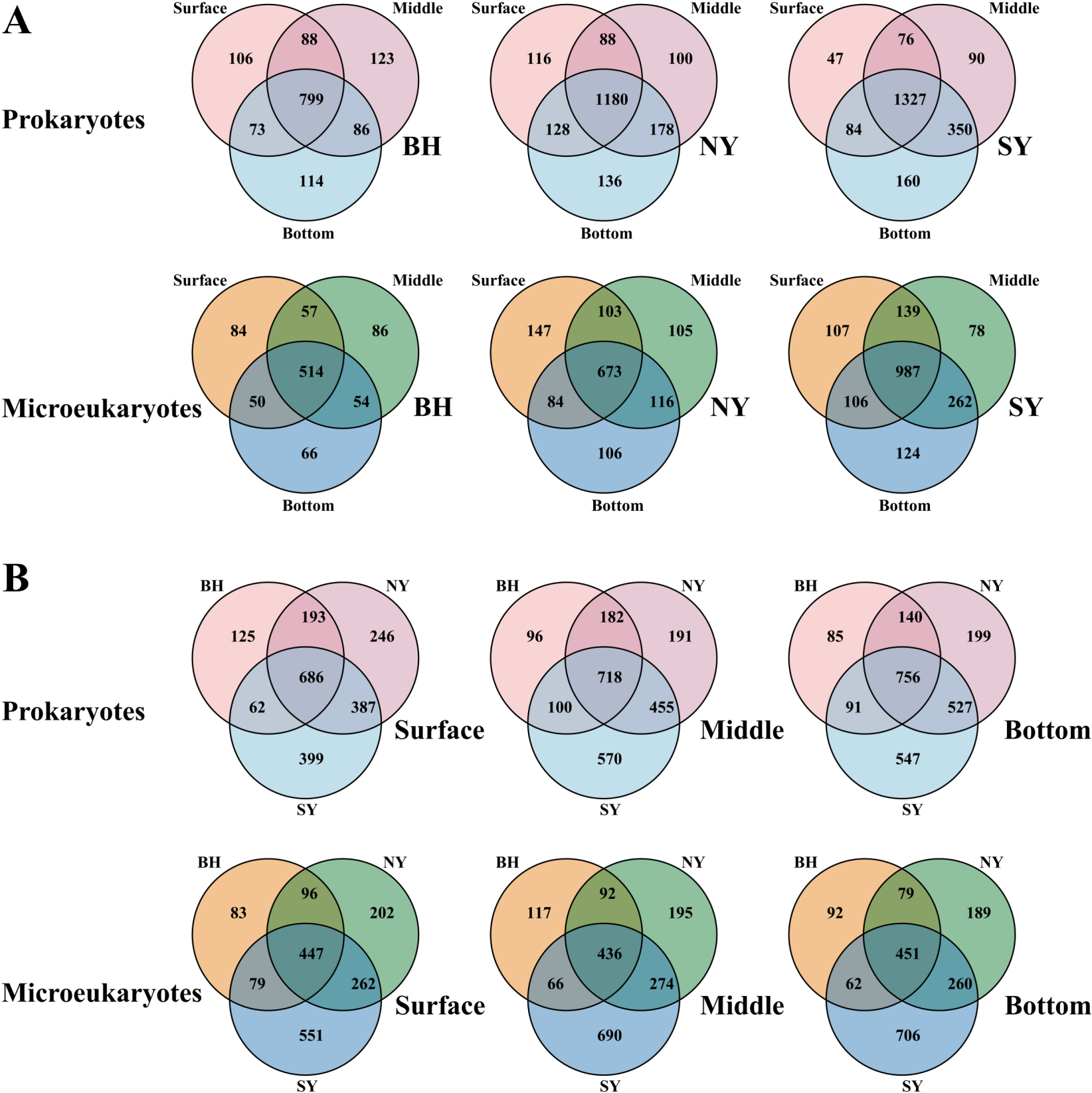


**FIG S11** Distribution of shared and unique microbial ASVs across spatial scales. (A) Comparison among water layers within each region. (B) Comparison among regions within each water layer.

**TABLE S1** Classification of water layers at each sampling station in the Bohai–Yellow Sea based on vertical density gradients, with the pycnocline defined as the depth interval where the density gradient continuously exceeds 0.01 kg m⁻³ m⁻¹.

| **Station** | **Sea area** | **Pycnocline** | **Surface** | **Middle** | **Bottom** |
| --- | --- | --- | --- | --- | --- |
| **B01** | BH | NO | 0 - 7.5 m | 7.5 - 17.5 m | 17.5 - 25 m |
| **B02** | BH | YES | 0 - 6 m | 6 - 15 m | 15 - 18 m |
| **B03** | BH | YES | 0 - 6 m | 6 - 7 m | 7 - 22 m |
| **B04** | BH | YES | 0 - 3 m | 3 - 13 m | 13 - 25 m |
| **B05** | BH | YES | 0 - 3 m | 3 - 8 m | 8 - 21 m |
| **N01** | NY | YES | 0 - 14 m | 14 - 15 m | 15 - 16 m |
| **N02** | NY | YES | 0 - 7 m | 7 - 56 m | 56 - 56 m |
| **N03** | NY | YES | 0 - 12 m | 12 - 16 m | 16 - 18 m |
| **N04** | NY | YES | 0 - 11 m | 11 - 17 m | 17 - 52 m |
| **N05** | NY | NO | 0 - 15.3 m | 15.3 - 35.7 m | 35.7 - 51 m |
| **N06** | NY | YES | 0 - 6 m | 6 - 34 m | 34 - 61 m |
| **N07** | NY | YES | 0 - 11 m | 11 - 30 m | 30 - 64 m |
| **S01** | SY | YES | 0 - 4 m | 4 - 7 m | 7 - 23 m |
| **S02** | SY | NO | 0 - 9.9 m | 9.9 - 23.1 m | 23.1 - 33 m |
| **S03** | SY | NO | 0 - 8.7 m | 8.7 - 20.3 m | 20.3 - 29 m |
| **S04** | SY | YES | 0 - 11 m | 11 - 11 m | 11 - 35 m |
| **S05** | SY | NO | 0 - 9 m | 9 - 21 m | 21 - 30 m |
| **S06** | SY | YES | 0 - 28 m | 28 - 47 m | 47 - 50 m |
| **S07** | SY | YES | 0 - 9 m | 9 - 38 m | 38 - 63 m |
| **S08** | SY | YES | 0 - 24 m | 24 - 29 m | 29 - 46 m |
| **S09** | SY | YES | 0 - 23 m | 23 - 60 m | 60 - 74 m |
| **S10** | SY | YES | 0 - 8 m | 8 - 54 m | 54 - 67 m |
| **S11** | SY | YES | 0 - 13 m | 13 - 51 m | 51 - 77 m |

**TABLE S2** Associations of horizontal and vertical gradients with microbial distribution in seawater. BHS/NYS/SYS represents the surface layer; BHM/NYM/SYM represents the middle layer; BHB/NYB/SYB represents the bottom layer. * indicates the significant correlation between microbial diversity and environmental factors (*, *p* < 0.05, **, *p* < 0.01, ***, *p* < 0.001).

|  | **Prokaryotes** | | | | | | **Microeukaryotes** | | | | | |
| --- | --- | --- | --- | --- | --- | --- | --- | --- | --- | --- | --- | --- |
| **Layer** | **MRPP** | | **ANOSIM** | | **Adonis** | | **MRPP** | | **ANOSIM** | | **Adonis** | |
|  | **A** | ***p*** | **r** | ***p*** | **F** | ***p*** | **A** | ***p*** | **r** | ***p*** | **F** | ***p*** |
| **All** | 0.198 | 0.001*** | 0.468 | 0.001*** | 4.920 | 0.001*** | 0.141 | 0.001*** | 0.371 | 0.001*** | 3.326 | 0.001*** |
| **BHS/SYS** | 0.091 | 0.002** | 0.408 | 0.022* | 3.601 | 0.002** | 0.056 | 0.004** | 0.523 | 0.007** | 2.910 | 0.005** |
| **BHS/NYS** | 0.074 | 0.022* | 0.399 | 0.017* | 2.772 | 0.015* | 0.050 | 0.008** | 0.493 | 0.004** | 2.269 | 0.006** |
| **BHS/BHM** | -0.069 | 0.969 | -0.176 | 0.960 | 0.200 | 0.953 | -0.072 | 0.993 | -0.144 | 0.805 | 0.225 | 0.984 |
| **BHS/SYM** | 0.114 | 0.002** | 0.808 | 0.003** | 5.151 | 0.002** | 0.040 | 0.005** | 0.377 | 0.006** | 2.247 | 0.005** |
| **BHS/NYM** | 0.075 | 0.032* | 0.322 | 0.023* | 2.912 | 0.016* | 0.059 | 0.011* | 0.540 | 0.004** | 2.474 | 0.010** |
| **BHS/BHB** | -0.050 | 0.952 | -0.092 | 0.836 | 0.428 | 0.952 | -0.055 | 0.993 | -0.128 | 0.83 | 0.342 | 0.984 |
| **BHS/SYB** | 0.120 | 0.002** | 0.732 | 0.003** | 5.313 | 0.004** | 0.038 | 0.011* | 0.408 | 0.002** | 2.318 | 0.008** |
| **BHS/NYB** | 0.084 | 0.016* | 0.408 | 0.011* | 2.946 | 0.015* | 0.060 | 0.012* | 0.574 | 0.006** | 2.542 | 0.011* |
| **SYS/NYS** | 0.087 | 0.002** | 0.400 | 0.005** | 4.036 | 0.004** | 0.044 | 0.004** | 0.413 | 0.001*** | 2.642 | 0.005** |
| **SYS/BHM** | 0.076 | 0.002** | 0.370 | 0.023* | 3.160 | 0.002** | 0.065 | 0.004** | 0.607 | 0.001*** | 3.192 | 0.005** |
| **SYS/SYM** | -0.004 | 0.722 | 0.001 | 0.524 | 0.962 | 0.542 | -0.009 | 0.976 | 0.017 | 0.387 | 0.853 | 0.755 |
| **SYS/NYM** | 0.097 | 0.002** | 0.442 | 0.005** | 4.520 | 0.002** | 0.065 | 0.004** | 0.557 | 0.001*** | 3.450 | 0.005** |
| **SYS/BHB** | 0.097 | 0.004** | 0.501 | 0.012* | 4.003 | 0.002** | 0.083 | 0.005** | 0.664 | 0.002** | 3.823 | 0.005** |
| **SYS/SYB** | 0.025 | 0.069 | 0.128 | 0.030* | 2.139 | 0.040* | 0.004 | 0.446 | 0.109 | 0.056 | 1.382 | 0.195 |
| **SYS/NYB** | 0.105 | 0.002** | 0.488 | 0.005** | 4.792 | 0.002** | 0.079 | 0.004** | 0.597 | 0.001*** | 4.102 | 0.005** |
| **NYS/BHM** | 0.068 | 0.022* | 0.388 | 0.021* | 2.654 | 0.021* | 0.051 | 0.011* | 0.438 | 0.006** | 2.298 | 0.013* |
| **NYS/SYM** | 0.111 | 0.002** | 0.616 | 0.003** | 5.815 | 0.002** | 0.024 | 0.015* | 0.191 | 0.03* | 1.758 | 0.013* |
| **NYS/NYM** | -0.038 | 0.969 | -0.136 | 0.969 | 0.316 | 0.953 | -0.021 | 0.993 | -0.047 | 0.645 | 0.608 | 0.984 |
| **NYS/BHB** | 0.081 | 0.013* | 0.458 | 0.017* | 2.977 | 0.017* | 0.062 | 0.010** | 0.445 | 0.005** | 2.564 | 0.013* |
| **NYS/SYB** | 0.113 | 0.002** | 0.570 | 0.003** | 5.834 | 0.002** | 0.036 | 0.004** | 0.384 | 0.001*** | 2.323 | 0.005** |
| **NYS/NYB** | -0.024 | 0.945 | -0.060 | 0.736 | 0.606 | 0.856 | -0.017 | 0.983 | 0.032 | 0.329 | 0.787 | 0.839 |
| **BM/SYM** | 0.100 | 0.002** | 0.787 | 0.005** | 4.551 | 0.004** | 0.045 | 0.005** | 0.377 | 0.006** | 2.331 | 0.005** |
| **BM/NYM** | 0.063 | 0.022* | 0.331 | 0.025* | 2.566 | 0.024* | 0.057 | 0.012* | 0.453 | 0.013* | 2.379 | 0.010** |
| **BHM/BHB** | -0.053 | 0.945 | -0.112 | 0.836 | 0.427 | 0.952 | -0.082 | 0.993 | -0.260 | 1.000 | 0.072 | 0.997 |
| **BHM/SYB** | 0.107 | 0.002** | 0.706 | 0.003** | 4.781 | 0.002** | 0.040 | 0.007** | 0.405 | 0.003** | 2.287 | 0.005** |
| **BHM/NYB** | 0.068 | 0.022* | 0.333 | 0.028* | 2.568 | 0.017* | 0.054 | 0.012* | 0.523 | 0.004** | 2.393 | 0.008** |
| **SYM/NYM** | 0.119 | 0.002** | 0.649 | 0.003** | 6.229 | 0.002** | 0.042 | 0.004** | 0.333 | 0.003** | 2.399 | 0.006** |
| **SYM/BHB** | 0.120 | 0.004** | 0.889 | 0.003** | 5.514 | 0.002** | 0.055 | 0.004** | 0.378 | 0.002** | 2.615 | 0.005** |
| **SYM/SYB** | -0.013 | 0.945 | -0.021 | 0.637 | 0.726 | 0.734 | -0.016 | 0.993 | -0.004 | 0.442 | 0.617 | 0.974 |
| **SYM/NYB** | 0.120 | 0.002** | 0.677 | 0.003** | 6.138 | 0.002** | 0.047 | 0.007** | 0.327 | 0.005** | 2.607 | 0.005** |
| **NYM/BHB** | 0.077 | 0.020* | 0.427 | 0.012* | 2.944 | 0.004** | 0.068 | 0.011* | 0.465 | 0.011* | 2.668 | 0.010** |
| **NYM/SYB** | 0.118 | 0.002** | 0.599 | 0.003** | 6.064 | 0.002** | 0.048 | 0.004** | 0.438 | 0.001*** | 2.705 | 0.005** |
| **NYM/NYB** | -0.042 | 0.969 | -0.128 | 0.960 | 0.283 | 0.953 | -0.052 | 0.993 | -0.155 | 0.95 | 0.088 | 0.997 |
| **BHB/SYB** | 0.116 | 0.002** | 0.814 | 0.003** | 5.192 | 0.002** | 0.052 | 0.004** | 0.438 | 0.002** | 2.649 | 0.005** |
| **BHB/NYB** | 0.070 | 0.017* | 0.357 | 0.017* | 2.617 | 0.017* | 0.061 | 0.012* | 0.499 | 0.005** | 2.559 | 0.013* |
| **SYB/NYB** | 0.108 | 0.002** | 0.561 | 0.003** | 5.518 | 0.002** | 0.050 | 0.005** | 0.412 | 0.001*** | 2.841 | 0.005** |

**TABLE S3** Keystone taxa within microbial co-occurrence networks of the Bohai-Yellow Sea.

| **Sea area** | **Taxa roles** | **Taxonomic** |
| --- | --- | --- |
| **Bohai Sea** | Module hubs | d__Bacteria; p__Bacteroidota; c__Bacteroidia; o__Flavobacteriales; f__Flavobacteriaceae; g__NS2b_marine_group |
|  | Module hubs | d__Bacteria; p__Proteobacteria; c__Gammaproteobacteria; o__Nitrosococcales; f__Nitrosococcaceae; g__Cm1-21; s__uncultured_gamma |
|  | Module hubs | d__Bacteria; p__Actinobacteriota; c__Actinobacteria; o__Micrococcales; f__Microbacteriaceae; g__Candidatus_Aquiluna |
|  | Module hubs | d__Bacteria; p__Proteobacteria; c__Gammaproteobacteria; o__Cellvibrionales; f__Spongiibacteraceae; g__BD1-7_clade |
|  | Module hubs | d__Eukaryota; p__Ochrophyta; c__Pelagophyceae; o__Pelagomonadales; f__Pelagomonadales; g__Aureococcus; s__Aureococcus_anophagefferens |
|  | Module hubs | d__Eukaryota; p__Ciliophora; c__Intramacronucleata; o__Spirotrichea |
|  | Connectors | d__Bacteria; p__Proteobacteria; c__Gammaproteobacteria; o__Cellvibrionales; f__Porticoccaceae; g__Porticoccus |
|  | Connectors | d__Bacteria; p__Proteobacteria; c__Alphaproteobacteria; o__Defluviicoccales; f__uncultured; g__uncultured |
|  | Connectors | d__Bacteria; p__Proteobacteria; c__Alphaproteobacteria; o__SAR11_clade |
|  | Connectors | d__Bacteria; p__Proteobacteria; c__Gammaproteobacteria; o__Steroidobacterales; f__Woeseiaceae; g__Woeseia; s__uncultured_marine |
|  | Connectors | d__Eukaryota; p__Ciliophora; c__Intramacronucleata; o__Spirotrichea |
|  | Connectors | d__Eukaryota; p__Dinoflagellata; c__Dinophyceae; o__Gymnodiniphycidae; f__Gymnodinium_clade |
| **North Yellow Sea** | Network hubs | d__Eukaryota; p__Ochrophyta; c__Pelagophyceae; o__Sarcinochrysidales; f__Sarcinochrysidales; g__Sarcinochrysidales; s__Pelagophyceae_sp. |
|  | Module hubs | d__Bacteria; p__Proteobacteria; c__Gammaproteobacteria; o__Burkholderiales; f__Methylophilaceae; g__OM43_clade |
|  | Module hubs | d__Bacteria; p__Proteobacteria; c__Alphaproteobacteria; o__SAR11_clade |
|  | Module hubs | d__Eukaryota; p__Chlorophyta; c__Mamiellophyceae; o__DSGM-81; f__DSGM-81; g__DSGM-81 |
|  | Connectors | d__Bacteria; p__Proteobacteria; c__Alphaproteobacteria; o__Rhodobacterales; f__Rhodobacteraceae |
|  | Connectors | d__Bacteria; p__Proteobacteria; c__Alphaproteobacteria; o__Rhodospirillales; f__AEGEAN-169_marine_group; g__AEGEAN-169_marine_group; s__uncultured_Alphaproteobacteria |
|  | Connectors | d__Bacteria; p__Proteobacteria; c__Gammaproteobacteria |
|  | Connectors | d__Bacteria; p__Proteobacteria; c__Alphaproteobacteria; o__Rhodobacterales; f__Rhodobacteraceae; g__Planktomarina |
|  | Connectors | d__Bacteria; p__Proteobacteria; c__Alphaproteobacteria; o__SAR11_clade; f__Clade_II; g__Clade_II |
|  | Connectors | d__Bacteria; p__Proteobacteria; c__Gammaproteobacteria; o__Oceanospirillales; f__Pseudohongiellaceae; g__Pseudohongiella |
|  | Connectors | d__Bacteria; p__Proteobacteria; c__Alphaproteobacteria; o__Rhodobacterales; f__Rhodobacteraceae |
|  | Connectors | d__Bacteria; p__Proteobacteria; c__Alphaproteobacteria; o__Puniceispirillales; f__SAR116_clade; g__SAR116_clade |
| **South Yellow Sea** | Network hubs | d__Eukaryota; p__Dinoflagellata; c__Dinophyceae |
|  | Module hubs | d__Bacteria; p__Proteobacteria; c__Alphaproteobacteria; o__Rhodospirillales; f__AEGEAN-169_marine_group; g__AEGEAN-169_marine_group |
|  | Module hubs | d__Bacteria; p__Proteobacteria; c__Gammaproteobacteria; o__uncultured; f__uncultured; g__uncultured; s__uncultured_Sinobacteraceae |
|  | Connectors | d__Bacteria; p__Proteobacteria; c__Gammaproteobacteria; o__SAR86_clade; f__SAR86_clade; g__SAR86_clade; s__uncultured_Oceanospirillales |
|  | Connectors | d__Bacteria; p__Proteobacteria; c__Gammaproteobacteria; o__Thiomicrospirales; f__Thioglobaceae; g__SUP05_cluster |
|  | Connectors | d__Eukaryota; p__Protalveolata; c__Syndiniales; o__Syndiniales; f__Syndiniales_Group_I; g__Syndiniales_Group_I |

**TABLE S4** Topological properties of microbial co-occurrence networks in the Bohai-Yellow Sea.

| **Sea area** | **Nodes** | **Edges** | **Average degree** | **Network diameter** | **Graph density** | **Modularity** | **Components** | **Average Clustering Coefficient** | **Average path length** | **Number of Clusters** | **Fragmentation (*f*)** |
| --- | --- | --- | --- | --- | --- | --- | --- | --- | --- | --- | --- |
| **BH** | 383 | 688 | 3.593 | 17 | 0.009 | 0.783 | 29 | 0.307 | 6.147 | 39 | 0.566119035 |
| **NY** | 343 | 886 | 5.166 | 12 | 0.015 | 0.581 | 41 | 0.301 | 4.289 | 49 | 0.636132843 |
| **SY** | 287 | 575 | 4.007 | 16 | 0.014 | 0.648 | 38 | 0.362 | 5.276 | 46 | 0.642741866 |

**TABLE S5** Comparison of network indices between observed microbial networks and 1000 degree-preserving random networks in the Bohai Sea and Yellow Sea. (*, *p* < 0.05; **, *p* < 0.01; ***, *p* < 0.001).

| **Topological indices** | **Network Indexes** | | | **Random Network Indexes (Mean +/- SD)** | | | **Z-score** | | | ***p*** | | | **q** | | |
| --- | --- | --- | --- | --- | --- | --- | --- | --- | --- | --- | --- | --- | --- | --- | --- |
|  | **BH** | **NY** | **SY** | **BH** | **NY** | **SY** | **BH** | **NY** | **SY** | **BH** | **NY** | **SY** | **BH** | **NY** | **SY** |
| **Centralization of degree** | 0.032 | 0.087 | 0.091 | 0.032 +/- 0 | 0.037 +/- 0 | 0.091 +/- 0 | - | - | - | - | - | - | - | - | - |
| **Density** | 0.009 | 0.015 | 0.014 | 0.009 +/- 0 | 0.006 +/- 0 | 0.014 +/- 0 | - | - | - | - | - | - | - | - | - |
| **Average clustering coefficient** | 0.208 | 0.181 | 0.213 | 0.004 +/- 0.003 | 0 +/- 0.001 | 0.012 +/- 0.006 | 69.818 | 36.472 | 36.308 | 0.010** | 0.010** | 0.010** | 0.011 | 0.011 | 0.011 |
| **Average path distance** | 6.147 | 4.289 | 5.276 | 4.389 +/- 0.047 | 6.555 +/- 0.281 | 3.676 +/- 0.052 | 37.800 | 19.242 | 30.743 | 0.010** | 0.010** | 0.010** | 0.011 | 0.011 | 0.011 |
| **Centralization of betweenness** | 0.108 | 0.064 | 0.187 | 0.071 +/- 0.010 | 0.178 +/- 0.04 | 0.118 +/- 0.014 | 3.792 | -1.902 | 4.955 | 0.010** | 0.980 | 0.010** | 0.011 | 1.000 | 0.011 |
| **Centralization of eigenvector centrality** | 0.963 | 0.923 | 0.921 | 0.856 +/- 0.018 | 0.959 +/- 0.009 | 0.881 +/- 0.010 | 5.910 | 5.941 | 3.921 | 0.010** | 0.010** | 0.010** | 0.011 | 0.011 | 0.011 |
| **Centralization of stress centrality** | 0.522 | 0.489 | 0.979 | 0.083 +/- 0.029 | 0.002+/- 0.002 | 0.116 +/- 0.033 | 14.917 | 16.424 | 25.816 | 0.010** | 0.010** | 0.010** | 0.011 | 0.011 | 0.011 |
| **Connectedness** | 0.668 | 0.507 | 0.427 | 0.907 +/- 0.027 | 0.619 +/- 0.046 | 0.874 +/- 0.031 | -8.985 | -14.114 | -14.603 | 0.010** | 0.010** | 0.010** | 0.011 | 0.011 | 0.011 |
| **Geodesic efficiency** | 0.198 | 0.284 | 0.263 | 0.254 +/- 0.002 | 0.184 +/- 0.006 | 0.304 +/- 0.003 | -25.206 | -11.891 | -12.358 | 0.010** | 0.010** | 0.010** | 0.011 | 0.011 | 0.011 |
| **Modularity** | 0.767 | 0.529 | 0.599 | 0.543 +/- 0.006 | 0.798 +/- 0.009 | 0.472 +/- 0.007 | 37.274 | 22.347 | 18.697 | 0.010** | 0.010** | 0.010** | 0.011 | 0.011 | 0.011 |
| **Transitivity** | 0.311 | 0.397 | 0.35 | 0.019 +/- 0.004 | 0.008 +/- 0.005 | 0.075 +/- 0.008 | 74.629 | 53.493 | 33.923 | 0.010** | 0.010** | 0.010** | 0.011 | 0.011 | 0.011 |

**TABLE S6**  Environmental variables of sampled stations in the Bohai–Yellow Sea.

| **Sample** | **Sea area** | **Layer** | **Longitude** | **Latitude** | **Distance to coastline** | **Depth** | **Chla** | **NO_3_^-^** | **PO_4_^3-^** | **NO_2_^-^** | **NH_4_^+^** | **SiO_3_^2-^** | **Temperature** | **Dissolved oxygen** | **Turbidity** | **ph** | **Salinity** | **Density** |
| --- | --- | --- | --- | --- | --- | --- | --- | --- | --- | --- | --- | --- | --- | --- | --- | --- | --- | --- |
| **B01L0** | BH | Surface | 38.354 | 120.748 | 7.405 | 3.300 | 1.810 | 7.140 | 0.680 | 0.100 | 2.620 | 4.390 | 5.420 | 288.470 | 1.690 | 12.770 | 31.158 | 1024.600 |
| **B01L1** | BH | Middle | 38.354 | 120.748 | 7.405 | 14.600 | 1.950 | 4.080 | 0.650 | 0.080 | 2.310 | 4.140 | 5.430 | 291.770 | 1.760 | 12.750 | 31.161 | 1024.656 |
| **B01L2** | BH | Bottom | 38.354 | 120.748 | 7.405 | 24.700 | 2.010 | 3.240 | 0.720 | 0.090 | 2.530 | 3.550 | 5.410 | 292.945 | 1.930 | 12.658 | 31.175 | 1024.717 |
| **B02L0** | BH | Surface | 38.335 | 119.012 | 21.042 | 3.110 | 1.550 | 11.100 | 0.480 | 0.180 | 1.810 | 8.480 | 7.617 | 274.977 | 5.574 | 10.310 | 29.626 | 1023.123 |
| **B02L1** | BH | Middle | 38.335 | 119.012 | 21.042 | 10.300 | 1.700 | 12.510 | 0.630 | 0.160 | 1.780 | 9.450 | 7.512 | 276.640 | 7.090 | 10.269 | 29.725 | 1023.247 |
| **B02L2** | BH | Bottom | 38.335 | 119.012 | 21.042 | 17.620 | 1.780 | 12.800 | 0.610 | 0.170 | 2.130 | 10.040 | 7.401 | 275.700 | 11.160 | 10.210 | 29.813 | 1023.368 |
| **B03L0** | BH | Surface | 38.969 | 118.959 | 16.590 | 2.810 | 2.540 | 1.600 | 1.260 | 0.070 | 1.770 | 1.040 | 7.772 | 282.915 | 1.532 | 10.694 | 30.416 | 1023.722 |
| **B03L1** | BH | Middle | 38.969 | 118.959 | 16.590 | 13.030 | 5.400 | 1.660 | 0.770 | 0.110 | 1.630 | 0.980 | 7.474 | 288.602 | 2.781 | 10.654 | 30.437 | 1023.825 |
| **B03L2** | BH | Bottom | 38.969 | 118.959 | 16.590 | 21.620 | 6.210 | 1.530 | 0.750 | 0.040 | 1.650 | 0.360 | 7.473 | 288.160 | 2.020 | 10.580 | 30.438 | 1023.868 |
| **B04L0** | BH | Surface | 39.602 | 120.618 | 60.984 | 3.130 | 1.800 | 2.770 | 0.690 | 0.110 | 1.430 | 1.190 | 5.890 | 300.960 | 0.980 | 10.940 | 30.147 | 1023.747 |
| **B04L1** | BH | Middle | 39.602 | 120.618 | 60.984 | 14.320 | 1.890 | 2.380 | 0.720 | 0.100 | 2.510 | 0.930 | 5.735 | 294.040 | 1.990 | 10.920 | 30.478 | 1024.078 |
| **B04L2** | BH | Bottom | 39.602 | 120.618 | 60.984 | 24.600 | 2.120 | 2.300 | 0.720 | 0.070 | 1.650 | 0.880 | 5.722 | 292.227 | 4.087 | 10.854 | 30.486 | 1024.137 |
| **B05L0** | BH | Surface | 38.868 | 119.753 | 69.633 | 3.130 | 1.950 | 5.560 | 0.810 | 0.100 | 1.510 | 2.300 | 7.420 | 287.570 | 2.080 | 10.520 | 30.605 | 1023.917 |
| **B05L1** | BH | Middle | 38.868 | 119.753 | 69.633 | 13.000 | 5.390 | 5.240 | 0.770 | 0.110 | 4.230 | 2.190 | 6.710 | 287.030 | 3.080 | 10.550 | 30.606 | 1024.057 |
| **B05L2** | BH | Bottom | 38.868 | 119.753 | 69.633 | 21.500 | 6.020 | 4.690 | 0.710 | 0.120 | 1.460 | 1.660 | 6.709 | 286.170 | 4.650 | 10.490 | 30.606 | 1024.094 |
| **N01L0** | NY | Surface | 37.898 | 121.198 | 19.540 | 3.480 | 2.780 | 2.590 | 0.680 | 0.100 | 1.430 | 1.420 | 8.290 | 271.900 | 3.820 | 9.760 | 30.478 | 1023.699 |
| **N01L1** | NY | Middle | 37.898 | 121.198 | 19.540 | 10.470 | 2.780 | 2.720 | 0.670 | 0.100 | 1.570 | 0.610 | 8.280 | 271.100 | 3.680 | 9.750 | 30.478 | 1023.732 |
| **N01L2** | NY | Bottom | 37.898 | 121.198 | 19.540 | 16.520 | 2.890 | 2.760 | 0.670 | 0.100 | 1.980 | 1.030 | 8.290 | 272.430 | 3.830 | 9.610 | 30.481 | 1023.760 |
| **N02L0** | NY | Surface | 38.529 | 121.379 | 26.168 | 3.420 | 1.650 | 0.330 | 0.500 | 0.070 | 1.620 | 1.540 | 7.440 | 289.580 | 0.650 | 11.110 | 30.983 | 1024.212 |
| **N02L1** | NY | Middle | 38.529 | 121.379 | 26.168 | 30.290 | 2.650 | 0.210 | 0.700 | 0.060 | 3.180 | 3.660 | 5.380 | 282.620 | 1.140 | 11.150 | 31.084 | 1024.671 |
| **N02L2** | NY | Bottom | 38.529 | 121.379 | 26.168 | 55.800 | 2.640 | 0.240 | 0.670 | 0.060 | 2.810 | 2.890 | 5.015 | 279.443 | 1.210 | 10.982 | 31.173 | 1024.903 |
| **N03L0** | NY | Surface | 39.569 | 123.894 | 22.630 | 3.500 | 2.610 | 0.680 | 0.590 | 0.120 | 1.880 | 1.180 | 6.600 | 280.960 | 0.640 | 10.720 | 29.324 | 1023.015 |
| **N03L1** | NY | Middle | 39.569 | 123.894 | 22.630 | 11.700 | 3.860 | 0.460 | 0.560 | 0.100 | 2.290 | 2.180 | 6.500 | 284.500 | 0.710 | 10.690 | 29.327 | 1023.078 |
| **N03L2** | NY | Bottom | 39.569 | 123.894 | 22.630 | 18.500 | 4.020 | 1.160 | 0.590 | 0.090 | 1.810 | 1.330 | 6.270 | 284.240 | 0.760 | 10.670 | 29.496 | 1023.259 |
| **N04L0** | NY | Surface | 38.836 | 123.319 | 24.226 | 3.510 | 1.130 | 0.060 | 0.570 | 0.040 | 1.520 | 5.430 | 8.030 | 291.020 | 0.360 | 11.270 | 31.098 | 1024.222 |
| **N04L1** | NY | Middle | 38.836 | 123.319 | 24.226 | 28.220 | 7.260 | 0.790 | 0.690 | 0.070 | 2.010 | 3.320 | 5.560 | 282.760 | 0.640 | 11.300 | 31.677 | 1025.110 |
| **N04L2** | NY | Bottom | 38.836 | 123.319 | 24.226 | 52.090 | 7.920 | 1.780 | 0.720 | 0.080 | 2.140 | 3.200 | 5.523 | 282.250 | 0.860 | 11.181 | 31.676 | 1025.226 |
| **N05L0** | NY | Surface | 38.820 | 123.809 | 65.433 | 3.030 | 5.090 | 3.350 | 0.750 | 0.140 | 1.380 | 2.300 | 7.060 | 286.110 | 0.770 | 10.060 | 31.900 | 1024.983 |
| **N05L1** | NY | Middle | 38.820 | 123.809 | 65.433 | 26.670 | 5.630 | 3.610 | 0.810 | 0.120 | 1.290 | 2.710 | 6.910 | 285.410 | 0.980 | 10.050 | 31.899 | 1025.110 |
| **N05L2** | NY | Bottom | 38.820 | 123.809 | 65.433 | 49.990 | 7.600 | 3.060 | 0.690 | 0.100 | 1.210 | 1.750 | 6.906 | 286.528 | 1.642 | 10.025 | 31.899 | 1025.222 |
| **N06L0** | NY | Surface | 38.083 | 123.209 | 84.636 | 2.900 | 2.760 | 0.430 | 0.400 | 0.180 | 1.230 | 0.840 | 9.490 | 288.770 | 0.640 | 10.070 | 31.804 | 1024.554 |
| **N06L1** | NY | Middle | 38.083 | 123.209 | 84.636 | 32.400 | 1.750 | 3.170 | 0.500 | 0.060 | 0.990 | 9.820 | 7.480 | 273.996 | 0.640 | 10.050 | 31.768 | 1024.957 |
| **N06L2** | NY | Bottom | 38.083 | 123.209 | 84.636 | 61.400 | 2.930 | 2.930 | 0.500 | 0.050 | 0.950 | 5.050 | 6.274 | 269.065 | 2.959 | 10.092 | 31.791 | 1025.269 |
| **N07L0** | NY | Surface | 37.518 | 123.249 | 56.020 | 3.200 | 0.760 | 0.080 | 0.460 | 0.020 | 0.950 | 0.650 | 8.960 | 277.630 | 0.610 | 9.850 | 31.020 | 1024.022 |
| **N07L1** | NY | Middle | 37.518 | 123.249 | 56.020 | 34.230 | 1.080 | 1.510 | 0.570 | 0.060 | 1.120 | 3.790 | 8.050 | 253.890 | 1.220 | 9.795 | 31.979 | 1025.051 |
| **N07L2** | NY | Bottom | 37.518 | 123.249 | 56.020 | 64.410 | 1.890 | 1.640 | 0.610 | 0.040 | 0.900 | 4.110 | 8.071 | 252.475 | 4.713 | 9.725 | 31.984 | 1025.191 |
| **S01L0** | SY | Surface | 36.500 | 122.005 | 35.283 | 3.070 | 1.710 | 0.550 | 0.260 | 0.090 | 0.880 | 0.900 | 10.200 | 255.500 | 3.150 | 10.510 | 30.740 | 1023.609 |
| **S01L1** | SY | Middle | 36.500 | 122.005 | 35.283 | 13.200 | 1.680 | 0.420 | 0.600 | 0.010 | 2.950 | 2.070 | 9.950 | 255.980 | 5.130 | 10.490 | 30.752 | 1023.704 |
| **S01L2** | SY | Bottom | 36.500 | 122.005 | 35.283 | 22.800 | 1.910 | 0.460 | 0.600 | 0.040 | 1.680 | 2.260 | 9.922 | 255.223 | 7.599 | 10.436 | 30.758 | 1023.760 |
| **S02L0** | SY | Surface | 35.971 | 121.023 | 15.703 | 2.600 | 1.290 | 0.140 | 0.660 | 0.070 | 1.970 | 2.260 | 10.135 | 247.756 | 1.647 | 10.418 | 31.011 | 1023.830 |
| **S02L1** | SY | Middle | 35.971 | 121.023 | 15.703 | 18.700 | 1.300 | 0.120 | 0.600 | 0.040 | 2.270 | 2.300 | 10.146 | 252.740 | 2.776 | 10.312 | 31.014 | 1023.904 |
| **S02L2** | SY | Bottom | 35.971 | 121.023 | 15.703 | 32.900 | 1.390 | 0.070 | 0.790 | 0.040 | 1.700 | 2.340 | 10.148 | 251.920 | 3.907 | 10.255 | 31.013 | 1023.967 |
| **S03L0** | SY | Surface | 35.006 | 120.508 | 73.402 | 3.100 | 1.960 | 0.250 | 0.600 | 0.050 | 1.270 | 2.290 | 10.693 | 248.983 | 1.500 | 10.826 | 31.029 | 1023.751 |
| **S03L1** | SY | Middle | 35.006 | 120.508 | 73.402 | 15.520 | 2.060 | 1.020 | 0.620 | 0.060 | 2.130 | 2.550 | 10.680 | 254.080 | 1.600 | 10.790 | 31.028 | 1023.808 |
| **S03L2** | SY | Bottom | 35.006 | 120.508 | 73.402 | 29.050 | 2.100 | 0.450 | 0.600 | 0.070 | 1.690 | 2.470 | 10.680 | 253.440 | 2.220 | 10.720 | 31.028 | 1023.873 |
| **S04L0** | SY | Surface | 33.984 | 122.328 | 173.731 | 3.510 | 1.190 | 0.050 | 0.570 | 0.040 | 1.480 | 3.030 | 11.390 | 249.970 | 0.780 | 9.380 | 31.680 | 1024.137 |
| **S04L1** | SY | Middle | 33.984 | 122.328 | 173.731 | 19.500 | 1.260 | 0.310 | 0.580 | 0.050 | 1.810 | 3.050 | 11.290 | 247.930 | 2.100 | 9.370 | 31.730 | 1024.265 |
| **S04L2** | SY | Bottom | 33.984 | 122.328 | 173.731 | 35.200 | 1.180 | 2.210 | 0.650 | 0.040 | 1.590 | 2.710 | 11.280 | 248.244 | 2.835 | 9.273 | 31.737 | 1024.346 |
| **S05L0** | SY | Surface | 32.984 | 122.999 | 158.494 | 3.480 | 1.190 | 5.010 | 0.820 | 0.420 | 1.380 | 11.440 | 12.340 | 232.810 | 5.150 | 9.690 | 32.169 | 1024.342 |
| **S05L1** | SY | Middle | 32.984 | 122.999 | 158.494 | 16.660 | 1.150 | 5.780 | 0.790 | 0.410 | 1.600 | 12.300 | 12.260 | 235.340 | 6.870 | 9.600 | 32.181 | 1024.423 |
| **S05L2** | SY | Bottom | 32.984 | 122.999 | 158.494 | 29.420 | 1.730 | 5.110 | 0.770 | 0.390 | 1.320 | 12.630 | 12.230 | 234.880 | 75.538 | 9.516 | 32.165 | 1024.481 |
| **S06L0** | SY | Surface | 34.991 | 121.952 | 139.924 | 4.800 | 1.230 | 0.150 | 0.590 | 0.060 | 1.700 | 1.720 | 9.835 | 266.094 | 0.527 | 10.047 | 30.712 | 1023.655 |
| **S06L1** | SY | Middle | 34.991 | 121.952 | 139.924 | 27.700 | 1.200 | 0.510 | 0.750 | 0.060 | 2.060 | 3.380 | 9.720 | 263.692 | 0.380 | 9.982 | 30.774 | 1023.828 |
| **S06L2** | SY | Bottom | 34.991 | 121.952 | 139.924 | 50.200 | 0.340 | 3.050 | 0.830 | 0.230 | 3.280 | 10.350 | 8.221 | 227.048 | 2.418 | 9.906 | 31.927 | 1025.060 |
| **S07L0** | SY | Surface | 33.988 | 122.997 | 208.493 | 3.030 | 0.490 | 0.140 | 0.590 | 0.040 | 2.080 | 3.600 | 11.756 | 250.695 | 0.334 | 9.484 | 32.162 | 1024.445 |
| **S07L1** | SY | Middle | 33.988 | 122.997 | 208.493 | 32.480 | 1.320 | 3.250 | 1.130 | 0.430 | 2.980 | 8.800 | 10.760 | 244.551 | 0.631 | 9.426 | 33.206 | 1025.568 |
| **S07L2** | SY | Bottom | 33.988 | 122.997 | 208.493 | 63.110 | 0.570 | 4.370 | 0.900 | 0.490 | 2.100 | 10.400 | 10.548 | 222.044 | 3.751 | 9.314 | 33.579 | 1026.035 |
| **S08L0** | SY | Surface | 32.980 | 123.987 | 154.842 | 4.000 | 5.170 | 0.750 | 0.660 | 0.070 | 1.610 | 11.200 | 12.340 | 248.740 | 0.970 | 9.690 | 32.843 | 1024.867 |
| **S08L1** | SY | Middle | 32.980 | 123.987 | 154.842 | 24.700 | 0.840 | 2.960 | 0.960 | 0.400 | 1.690 | 15.960 | 12.130 | 247.570 | 1.380 | 9.630 | 32.858 | 1025.040 |
| **S08L2** | SY | Bottom | 32.980 | 123.987 | 154.842 | 45.800 | 0.910 | 3.610 | 0.800 | 0.430 | 1.830 | 11.380 | 11.776 | 237.221 | 21.312 | 9.605 | 32.915 | 1025.220 |
| **S09L0** | SY | Surface | 36.506 | 123.997 | 145.168 | 3.400 | 0.690 | 0.330 | 0.560 | 0.070 | 1.570 | 1.030 | 11.130 | 263.280 | 0.370 | 9.630 | 32.342 | 1024.697 |
| **S09L1** | SY | Middle | 36.506 | 123.997 | 145.168 | 39.400 | 0.880 | 0.170 | 0.640 | 0.040 | 2.500 | 1.840 | 9.477 | 261.211 | 0.208 | 9.520 | 32.416 | 1025.204 |
| **S09L2** | SY | Bottom | 36.506 | 123.997 | 145.168 | 74.600 | 0.430 | 5.180 | 0.840 | 0.180 | 1.710 | 10.890 | 9.890 | 267.360 | 0.320 | 9.550 | 32.557 | 1025.561 |
| **S10L0** | SY | Surface | 35.856 | 122.996 | 114.641 | 3.570 | 1.480 | 0.800 | 0.660 | 0.060 | 2.230 | 2.440 | 10.490 | 258.132 | 0.377 | 10.403 | 32.072 | 1024.598 |
| **S10L1** | SY | Middle | 35.856 | 122.996 | 114.641 | 35.770 | 0.750 | 1.780 | 0.750 | 0.110 | 2.020 | 4.720 | 10.288 | 260.085 | 0.321 | 10.310 | 32.526 | 1025.137 |
| **S10L2** | SY | Bottom | 35.856 | 122.996 | 114.641 | 67.040 | 0.230 | 5.020 | 0.980 | 0.290 | 3.100 | 11.910 | 9.705 | 219.262 | 1.392 | 10.185 | 33.097 | 1025.822 |
| **S11L0** | SY | Surface | 34.995 | 123.978 | 124.346 | 2.900 | 0.350 | 0.380 | 0.640 | 0.050 | 1.840 | 1.890 | 11.309 | 252.940 | 0.285 | 10.166 | 32.509 | 1024.795 |
| **S11L1** | SY | Middle | 34.995 | 123.978 | 124.346 | 40.560 | 0.290 | 7.780 | 0.980 | 0.170 | 3.050 | 11.930 | 9.206 | 259.673 | 0.342 | 10.126 | 32.840 | 1025.583 |
| **S11L2** | SY | Bottom | 34.995 | 123.978 | 124.346 | 76.420 | 0.450 | 10.310 | 1.040 | 0.070 | 2.390 | 17.050 | 9.950 | 205.713 | 8.325 | 10.066 | 33.361 | 1026.028 |

**TABLE S7** Independent effects (adj.R2) of environmental variables on microbial community structure in the Bohai–Yellow Sea. Shared and total adj.R2 denote the joint contribution of all variables and the sum of unique and shared fractions, respectively. (*, *p* < 0.05, **, *p* < 0.01, ***, *p* < 0.001).

| **Sea area** | **Environmental variables** | **Prokaryotes** | | | **Microeukaryotes** | | |
| --- | --- | --- | --- | --- | --- | --- | --- |
|  |  | **Independent (adj.R^2^)** | **F** | ***p*** | **Independent (adj.R^2^)** | **F** | ***p*** |
| **All** | **Distance to coastline** | 0.015 | 2.441 | 0.009** | 0.022 | 2.809 | <0.001*** |
|  | **Depth** | 0.014 | 2.388 | 0.012* | 0.009 | 1.765 | 0.022* |
|  | **Chla** | 0.004 | 1.418 | 0.151 | 0.012 | 1.958 | 0.008** |
|  | **Temperature** | 0.092 | 9.975 | <0.001*** | 0.044 | 4.652 | <0.001*** |
|  | **Salinity** | 0.049 | 5.812 | <0.001*** | 0.025 | 3.057 | <0.001*** |
|  | **NO_3_^-^** | 0.036 | 4.502 | <0.001*** | 0.036 | 3.969 | <0.001*** |
|  | **PO_4_^3-^** | 0.016 | 2.519 | 0.009** | 0.015 | 2.207 | 0.002** |
|  | **NO_2_^-^** | 0.011 | 2.029 | 0.036* | 0.019 | 2.569 | <0.001*** |
|  | **Shared (adj.R^2^)** | **0.140** | - | - | **0.087** | - | - |
|  | **Total (adj.R^2^)** | **0.376** | - | - | **0.269** | - | - |
| **Bohai Sea** | **Distance to coastline** | 0.231 | 6.375 | <0.001*** | 0.342 | 11.966 | <0.001*** |
|  | **Depth** | 0.035 | 1.806 | 0.117 | -0.002 | 0.922 | 0.516 |
|  | **Chla** | -0.006 | 0.850 | 0.530 | 0.011 | 1.368 | 0.255 |
|  | **Temperature** | 0.028 | 1.645 | 0.162 | 0.025 | 1.810 | 0.110 |
|  | **Salinity** | 0.197 | 5.586 | <0.001*** | 0.228 | 8.315 | <0.001*** |
|  | **NO_3_^-^** | 0.074 | 2.714 | 0.016* | 0.085 | 3.724 | 0.005** |
|  | **PO_4_^3-^** | -0.010 | 0.775 | 0.595 | 0.003 | 1.085 | 0.392 |
|  | **NO_2_^-^** | -0.018 | 0.589 | 0.733 | 0.002 | 1.053 | 0.404 |
|  | **Shared (adj.R^2^)** | **0.340** | - | - | **0.088** | - | - |
|  | **Total (adj.R^2^)** | **0.689** | - | - | **0.782** | - | - |
| **North Yellow Sea** | **Distance to coastline** | 0.091 | 4.365 | <0.001*** | 0.090 | 3.691 | <0.001*** |
|  | **Depth** | 0.029 | 2.060 | 0.061 | 0.024 | 1.712 | 0.054 |
|  | **Chla** | 0.041 | 2.511 | 0.038* | 0.055 | 2.654 | 0.003** |
|  | **Temperature** | 0.099 | 4.699 | 0.003** | 0.048 | 2.445 | 0.003** |
|  | **Salinity** | 0.091 | 4.407 | 0.004** | 0.083 | 3.467 | 0.002** |
|  | **NO_3_^-^** | 0.031 | 2.161 | 0.058 | 0.078 | 3.333 | 0.002** |
|  | **PO_4_^3-^** | 0.032 | 2.179 | 0.052 | 0.054 | 2.597 | 0.004** |
|  | **NO_2_^-^** | 0.034 | 2.247 | 0.043* | 0.036 | 2.082 | 0.006** |
|  | **Shared (adj.R^2^)** | **0.202** | - | - | **0.097** | - | - |
|  | **Total (adj.R^2^)** | **0.650** | - | - | **0.565** | - | - |
| **South Yellow Sea** | **Distance to coastline** | 0.065 | 4.079 | <0.001*** | 0.057 | 3.323 | <0.001*** |
|  | **Depth** | 0.018 | 1.832 | 0.064 | -0.001 | 0.958 | 0.500 |
|  | **Chla** | 0.015 | 1.701 | 0.067 | 0.020 | 1.803 | 0.028* |
|  | **Temperature** | 0.029 | 2.382 | 0.010** | 0.028 | 2.141 | 0.009** |
|  | **Salinity** | 0.073 | 4.471 | <0.001*** | 0.052 | 3.101 | 0.002** |
|  | **NO_3_^-^** | 0.045 | 3.139 | 0.004** | 0.033 | 2.341 | 0.006** |
|  | **PO_4_^3-^** | 0.004 | 1.185 | 0.286 | -0.001 | 0.958 | 0.474 |
|  | **NO_2_^-^** | 0.031 | 2.471 | 0.008** | 0.044 | 2.788 | <0.001*** |
|  | **Shared (adj.R^2^)** | **0.195** | - | - | **0.154** | - | - |
|  | **Total (adj.R^2^)** | **0.475** | - | - | **0.386** | - | - |

**TABLE S8** Variance Inflation Factor (VIF) for environmental variables in the Bohai-Yellow Sea.

| **Environmental variables** | **Variance Inflation Factor** | | | |
| --- | --- | --- | --- | --- |
|  | **All** | **Bohai Sea** | **North Yellow Sea** | **South Yellow Sea** |
| **Distance to coastline** | 3.263 | 1.336 | 5.848 | 2.209 |
| **Depth** | 1.773 | 1.711 | 2.776 | 3.200 |
| **Chla** | 1.406 | 3.230 | 2.775 | 1.493 |
| **Temperature** | 2.797 | 6.620 | 3.299 | 2.720 |
| **Salinity** | 3.188 | 4.764 | 4.103 | 3.628 |
| **NO_3_^-^** | 1.435 | 6.216 | 3.337 | 2.980 |
| **PO_4_^3-^** | 1.505 | 3.179 | 4.912 | 3.551 |
| **NO_2_^-^** | 1.884 | 5.865 | 2.344 | 2.383 |

**TABLE S9** Connectivity of prokaryotic and eukaryotic microbiomes across spatial gradients in the Bohai-Yellow Sea. Comparisons include vertical (surface/middle/bottom), horizontal (BH/NY/SY), and nearshore-offshore gradients. "Filtered" indicates the removal of rare ASVs (relative abundance < 0.01% and occurrence < 10%). (*, *p* < 0.05, **, *p* < 0.01, ***, *p* < 0.001).

|  |  |  | **All** | | | | **Filtered (relative abundance < 0.01% and occurrence < 10%)** | | | |
| --- | --- | --- | --- | --- | --- | --- | --- | --- | --- | --- |
|  |  |  | **Connectivity (%)** | |  |  | **Connectivity (%)** | |  |  |
| **Type** | **Source** | **Target** | **Prokaryotes** | **Microeukaryotes** | **chi_sq** | ***p.*adj_BH** | **Prokaryotes** | **Microeukaryotes** | **chi_sq** | ***p*.adj_BH** |
| **Vertical** | BHS | BHM | 80.93 | 80.31 | 0.071 | 0.829 | 82.785 | 84.458 | 0.690 | 0.522 |
|  | BHS | BHB | 81.34 | 82.46 | 0.276 | 0.718 | 82.286 | 86.124 | 3.968 | 0.114 |
|  | BHM | BHB | 82.56 | 83.04 | 0.039 | 0.843 | 83.810 | 86.443 | 1.913 | 0.293 |
|  | NYS | NYM | 82.02 | 77.83 | 6.467 | 0.023* | 83.800 | 83.144 | 11.780 | 0.002** |
|  | NYS | NYB | 80.64 | 77.32 | 3.906 | 0.068 | 84.667 | 80.645 | 6.313 | 0.022* |
|  | NYM | NYB | 83.72 | 80.59 | 3.940 | 0.068 | 86.954 | 83.441 | 5.447 | 0.031* |
|  | SYS | SYM | 76.13 | 76.81 | 0.175 | 0.761 | 80.182 | 80.490 | 0.027 | 0.939 |
|  | SYS | SYB | 73.45 | 73.90 | 0.066 | 0.829 | 77.681 | 77.852 | 0.005 | 0.981 |
|  | SYM | SYB | 87.30 | 84.45 | 5.420 | 0.036* | 89.292 | 92.388 | 7.523 | 0.018* |
| **Horizontal** | BHS | NYS | 58.13 | 53.92 | 4.193 | 0.064 | 60.183 | 55.855 | 4.254 | 0.056* |
|  | BHS | SYS | 48.76 | 39.28 | 25.642 | < 0.001*** | 51.868 | 42.110 | 24.909 | < 0.001*** |
|  | NYS | SYS | 69.95 | 52.95 | 86.980 | < 0.001*** | 71.659 | 61.896 | 27.007 | < 0.001*** |
|  | BHM | NYM | 58.21 | 52.96 | 6.589 | 0.023* | 61.270 | 54.995 | 9.033 | 0.005** |
|  | BHM | SYM | 44.38 | 34.24 | 34.600 | < 0.001*** | 44.773 | 38.303 | 12.829 | < 0.001*** |
|  | NYM | SYM | 63.65 | 48.43 | 76.460 | < 0.001*** | 64.149 | 53.823 | 33.395 | < 0.001*** |
|  | BHB | NYB | 55.24 | 54.14 | 0.257 | 0.718 | 55.487 | 57.407 | 0.800 | 0.528 |
|  | BHB | SYB | 44.09 | 34.69 | 30.414 | < 0.001*** | 44.649 | 39.567 | 7.920 | 0.011* |
|  | NYB | SYB | 66.79 | 48.07 | 119.913 | < 0.001*** | 67.528 | 54.637 | 53.879 | < 0.001*** |
| **Distance to coastline** | Near-S | Mid-S | 66.71 | 71.07 | 5.503 | 0.036* | 66.833 | 75.271 | 20.717 | < 0.001*** |
|  | Near-S | Off-S | 64.90 | 62.23 | 1.723 | 0.256 | 65.379 | 66.802 | 0.451 | 0.663 |
|  | Mid-S | Off-S | 74.71 | 63.73 | 33.647 | < 0.001*** | 75.186 | 68.737 | 11.512 | 0.002** |
|  | Near-M | Mid-M | 71.70 | 69.38 | 1.576 | 0.269 | 72.206 | 73.313 | 0.332 | 0.663 |
|  | Near-M | Off-M | 60.72 | 51.57 | 24.280 | < 0.001*** | 61.049 | 57.058 | 4.313 | 0.079 |
|  | Mid-M | Off-M | 72.18 | 54.39 | 98.755 | < 0.001*** | 72.722 | 60.204 | 47.810 | < 0.001*** |
|  | Near-B | Mid-B | 73.43 | 69.35 | 5.291 | 0.036 | 73.936 | 73.013 | 0.236 | 0.705 |
|  | Near-B | Off-B | 60.40 | 53.20 | 15.348 | < 0.001*** | 65.338 | 57.571 | 16.890 | < 0.001*** |
|  | Mid-B | Off-B | 73.24 | 57.97 | 76.909 | < 0.001*** | 73.913 | 65.310 | 23.890 | < 0.001*** |
